# Ethnic differences in SARS-CoV-2 infection and COVID-19-related hospitalisation, intensive care unit admission, and death in 17 million adults in England: an observational cohort study using the OpenSAFELY platform

**DOI:** 10.1016/S0140-6736(21)00634-6

**Published:** 2021-05-08

**Authors:** Rohini Mathur, Christopher T Rentsch, Caroline E Morton, William J Hulme, Anna Schultze, Brian MacKenna, Rosalind M Eggo, Krishnan Bhaskaran, Angel Y S Wong, Elizabeth J Williamson, Harriet Forbes, Kevin Wing, Helen I McDonald, Chris Bates, Seb Bacon, Alex J Walker, David Evans, Peter Inglesby, Amir Mehrkar, Helen J Curtis, Nicholas J DeVito, Richard Croker, Henry Drysdale, Jonathan Cockburn, John Parry, Frank Hester, Sam Harper, Ian J Douglas, Laurie Tomlinson, Stephen J W Evans, Richard Grieve, David Harrison, Kathy Rowan, Kamlesh Khunti, Nishi Chaturvedi, Liam Smeeth, Ben Goldacre

**Affiliations:** a Department of Non-Communicable Disease Epidemiology, London School of Hygiene & Tropical Medicine, London, UK; b Department of Medical Statistics, London School of Hygiene & Tropical Medicine, London, UK; c Department of Infectious Disease Epidemiology, London School of Hygiene & Tropical Medicine, London, UK; d Department of Health Services Research and Policy, London School of Hygiene & Tropical Medicine, London, UK; e The DataLab, Nuffield Department of Primary Care Health Sciences, University of Oxford, Oxford, UK; f National Institute for Health Research Health Protection Research Unit in Immunisation, London, UK; g TPP, Leeds, UK; h Intensive Care National Audit and Research Centre, London, UK; i Diabetes Research Centre, University of Leicester, Leicester, UK; j Medical Research Council Unit for Lifelong Health and Ageing, University College London, London, UK

## Abstract

**Background:**

COVID-19 has disproportionately affected minority ethnic populations in the UK. Our aim was to quantify ethnic differences in SARS-CoV-2 infection and COVID-19 outcomes during the first and second waves of the COVID-19 pandemic in England.

**Methods:**

We conducted an observational cohort study of adults (aged ≥18 years) registered with primary care practices in England for whom electronic health records were available through the OpenSAFELY platform, and who had at least 1 year of continuous registration at the start of each study period (Feb 1 to Aug 3, 2020 [wave 1], and Sept 1 to Dec 31, 2020 [wave 2]). Individual-level primary care data were linked to data from other sources on the outcomes of interest: SARS-CoV-2 testing and positive test results and COVID-19-related hospital admissions, intensive care unit (ICU) admissions, and death. The exposure was self-reported ethnicity as captured on the primary care record, grouped into five high-level census categories (White, South Asian, Black, other, and mixed) and 16 subcategories across these five categories, as well as an unknown ethnicity category. We used multivariable Cox regression to examine ethnic differences in the outcomes of interest. Models were adjusted for age, sex, deprivation, clinical factors and comorbidities, and household size, with stratification by geographical region.

**Findings:**

Of 17 288 532 adults included in the study (excluding care home residents), 10 877 978 (62·9%) were White, 1 025 319 (5·9%) were South Asian, 340 912 (2·0%) were Black, 170 484 (1·0%) were of mixed ethnicity, 320 788 (1·9%) were of other ethnicity, and 4 553 051 (26·3%) were of unknown ethnicity. In wave 1, the likelihood of being tested for SARS-CoV-2 infection was slightly higher in the South Asian group (adjusted hazard ratio 1·08 [95% CI 1·07–1·09]), Black group (1·08 [1·06–1·09]), and mixed ethnicity group (1·04 [1·02–1·05]) and was decreased in the other ethnicity group (0·77 [0·76–0·78]) relative to the White group. The risk of testing positive for SARS-CoV-2 infection was higher in the South Asian group (1·99 [1·94–2·04]), Black group (1·69 [1·62–1·77]), mixed ethnicity group (1·49 [1·39–1·59]), and other ethnicity group (1·20 [1·14–1·28]). Compared with the White group, the four remaining high-level ethnic groups had an increased risk of COVID-19-related hospitalisation (South Asian group 1·48 [1·41–1·55], Black group 1·78 [1·67–1·90], mixed ethnicity group 1·63 [1·45–1·83], other ethnicity group 1·54 [1·41–1·69]), COVID-19-related ICU admission (2·18 [1·92–2·48], 3·12 [2·65–3·67], 2·96 [2·26–3·87], 3·18 [2·58–3·93]), and death (1·26 [1·15–1·37], 1·51 [1·31–1·71], 1·41 [1·11–1·81], 1·22 [1·00–1·48]). In wave 2, the risks of hospitalisation, ICU admission, and death relative to the White group were increased in the South Asian group but attenuated for the Black group compared with these risks in wave 1. Disaggregation into 16 ethnicity groups showed important heterogeneity within the five broader categories.

**Interpretation:**

Some minority ethnic populations in England have excess risks of testing positive for SARS-CoV-2 and of adverse COVID-19 outcomes compared with the White population, even after accounting for differences in sociodemographic, clinical, and household characteristics. Causes are likely to be multifactorial, and delineating the exact mechanisms is crucial. Tackling ethnic inequalities will require action across many fronts, including reducing structural inequalities, addressing barriers to equitable care, and improving uptake of testing and vaccination.

**Funding:**

Medical Research Council.

**Research in context**

**Evidence before this study**

We searched PubMed for population-based studies examining the association between ethnicity and COVID-19. Keywords included (ethnic* OR race) AND (COVID OR coronavirus OR SARS-CoV-2) AND (UK or England) AND (risk OR rate OR odds). Results were filtered to human studies published from 2019 onwards with abstracts available. We identified six studies examining ethnic differences in SARS-CoV-2 infection and COVID-19 outcomes in population-based samples. Five studies from the UK Biobank reported an increased risk of SARS-CoV-2 infection and COVID-19-related hospitalisation in Black and South Asian groups compared with White groups. As the UK Biobank cohort is known to be healthier, less deprived, and less ethnically diverse than the UK population, these findings are not wholly generalisable to the wider UK population. Our previous study using the OpenSAFELY platform showed an increased risk of COVID-19-related death in minority ethnic groups, but did not examine the role of household size or examine ethnic differences in SARS-CoV-2 infection and COVID-19 outcomes earlier in the care pathway.

**Added value of this study**

This is the largest study in the UK to examine ethnic inequalities in testing positive for SARS-CoV-2 and in COVID-19-related outcomes in a cohort covering 40% of the population in England. Additionally, it is the only population-representative study to date that accounts for household size in addition to sociodemographic characteristics and clinical comorbidities. By examining ethnicity according to both high-level and detailed ethnic groupings, we have shown important ethnic differences in the risk of testing positive for SARS-CoV-2 and the risks of COVID-19-related hospital admission, intensive care unit admissions, and death. We showed that multiple factors contribute to ethnic inequalities in COVID-19 and the importance of these factors varies by ethnic group. Compared with wave 1, the risks of COVID-19-related hospitalisation and death in wave 2 were increased for South Asian groups and reduced in all other ethnic minority groups relative to the White group.

**Implications of all the available evidence**

The risks of SARS-CoV-2 infection and severe COVID-19 outcomes are disproportionately increased in minority ethnic groups, both in the UK and internationally. Reducing ethnic inequalities in COVID-19 risks requires action on social determinants including addressing disadvantage and discrimination, reducing risk of infection and transmission, improving quality of and access to quality clinical care, and improving management of pre-existing clinical conditions. The appropriate balance of these actions needs tailoring for different ethnic groups.

## Introduction

The risks of SARS-CoV-2 infection and COVID-19 disease have been reported to be disproportionately increased in minority ethnic groups compared with White groups^1^, ^2^, ^3^, ^4^ in the UK and in other countries, including the USA^5^ and Brazil.^6^ It is hypothesised that these differences might be driven by factors such as living in deprived areas; working in high-exposure or front-line occupations; living in large, multigenerational households; a higher burden of underlying conditions; discrimination; and poor access to health or community services.^7^, ^8^, ^9^, ^10^

In the UK, the collection of ethnicity data is considered essential for identifying and reducing ethnic inequalities.^11^, ^12^ Although there is no single universally accepted definition of ethnicity, it serves as an important social construct and surrogate marker for shared exposures or risks for people with similar social, biological, and cultural characteristics.^13^, ^14^, ^15^

To date, many studies on COVID-19 have reported findings according to broad ethnic categories—such as White, South Asian, and Black—rather than considering disaggregated groupings. Furthermore, most evidence has been derived from populations with severe disease requiring hospitalisation, making it difficult to extrapolate findings to the general population.^16^, ^17^, ^18^, ^19^, ^20^, ^21^ Finally, although previous studies have accounted for health status, socioeconomic deprivation, or household composition, none yet have considered these factors in conjunction.^22^, ^23^

The aim of this study was to estimate the effect of ethnicity on being tested and testing positive for SARS-CoV-2, and on COVID-19-related hospitalisation, intensive care unit (ICU) admission, and mortality, recognising the potential role of sociodemographic, clinical, and household-related factors.

## Methods

### Study design and population

We did a population-based, observational cohort study using the OpenSAFELY platform, for which National Health Service (NHS) England is the data controller. OpenSAFELY holds electronic health record data for 24 million people registered with primary care practices using TPP software, representing around 40% of the population of England.

Individual-level primary care data were linked to SARS-CoV-2 testing data from the Second Generation Surveillance System, COVID-19-related hospital admissions from the Secondary Uses Service, COVID-19-related ICU admissions from the Intensive Care National Audit & Research Centre,^24^ and mortality data from the Office for National Statistics (ONS). The study population comprised adults aged 18 years and older who were registered with a primary care practice on Feb 1, 2020. The study periods were from Feb 1 to Aug 3, 2020 (for wave 1), and from Sept 1 to Dec 31, 2020 (for wave 2). A minimum of 12 months of continuous registration before the start date of each wave was required for inclusion, to ensure that baseline factors were adequately captured. Individuals residing in care homes were excluded from the main analyses because we hypothesised that the role of sociodemographic, clinical, and household characteristics would be systematically different for care home residents than for the general population.

This study was approved by the Health Research Authority (REC reference 20/LO/0651) and by the London School of Hygiene & Tropical Medicine's Ethics Board (reference 21863). Patient data were pseudonymised for analysis and linkage using industry standard cryptographic hashing techniques, and all pseudonymised datasets transmitted for linkage onto OpenSAFELY were encrypted. Only aggregate statistical outputs could leave the TPP platform environment, following best practice for anonymisation of results such as statistical disclosure control for low cell counts. The OpenSAFELY research platform adheres to the data protection principles of the UK Data Protection Act 2018 and the EU General Data Protection Regulation 2016.

### Exposures

The primary exposure was self-reported ethnicity as captured on the primary care record, collapsed into the five high-level and 16 detailed census categories of White (White British, White Irish, other White), South Asian (Indian, Pakistani, Bangladeshi, other South Asian), Black (African, Caribbean, other Black), other (Chinese, all others), and mixed (White and Asian, White and African, White and Caribbean, other mixed). An unknown ethnicity category was also included. Comparisons were reported for the five high-level ethnic groups with the White group as the reference category, and for the 16 disaggregated groups, with the White British group as the reference category.

### Outcomes

Outcomes of interest included receiving a PCR test for SARS-CoV-2, testing positive for SARS-CoV-2, and COVID-19-related hospital admission, ICU admission, and death (defined as the presence of ICD-10 codes U07.1 [confirmed COVID-19] or U07.2 [suspected COVID-19] anywhere on the death certificate). Testing outcomes were obtained from the UK's pillar 1 (NHS and Public Health England laboratories) and pillar 2 (commercial partners) testing strategies and included results from PCR swab tests used to identify symptomatic individuals.^25^, ^26^

### Covariates

Demographic characteristics included age, sex, deprivation, household size, number of primary care consultations in the past 12 months, and geographical region (defined by the sustainability and transformation partnership [STP], an NHS administrative area). Deprivation was defined using quintiles of the Index of Multiple Deprivation, an area-level composite measure of seven domains: income, employment, education, skills and training, health and disability, crime, and barriers to housing services and living environment.^27^ Household size (categorised as 1–2, 3–5, 6–10, or ≥11 people) was determined using the number of individuals of all ages in OpenSAFELY residing at the same address on Feb 1, 2020.

Clinical covariates were identified using the Read clinical classification system^28^ and included body-mass index (BMI), glycated haemoglobin (HbA_1c_), blood pressure, and smoking status. BMI in kg/m^2^ was grouped into six categories using the WHO classification, with adjustments for South Asian ethnicity: underweight (<18·5 kg/m^2^), normal weight (18·5–24·9 kg/m^2^ [or 18·5–23·5 kg/m^2^ if South Asian]), overweight (25·0–29·9 kg/m^2^ [23·6–27·5 kg/m^2^]); obese I (30·0–34·9 kg/m^2^ [27·5–32·4 kg/m^2^]); obese II (35·0–39·9 kg/m^2^ [32·5–37·4 kg/m^2^]); and obese III (≥40 kg/m^2^ [≥37·5 kg/m^2^]). HbA_1c_ was grouped into five categories (<6·5%, 6·5–7·4%, 7·5–7·9%, 8·0–8·9%, and ≥9·0%). Blood pressure was grouped into four categories: normal (<120/<80); elevated (120 to 130/<80); high, stage I (131 to <140/80 to <90); and high, stage II (≥140/≥90). Smoking status was grouped into current, former, and never smokers. Those with missing smoking status were categorised as never smokers. Those with missing BMI, HbA_1c,_ or blood pressure data were grouped into a separate category of “unknown”.

Clinical comorbidities were considered present at baseline if they were recorded at any time before Feb 1, 2020 (for wave 1) or Sept 1, 2020 (for wave 2). Comorbidities included the following: hypertension, asthma, chronic respiratory disease, chronic heart disease, type 1 and type 2 diabetes, cancer, chronic liver disease, stroke, dementia, other chronic neurological diseases, chronic kidney disease (defined as an estimated glomerular filtration rate <60 mL/min per 1·73 m^2^), end-stage renal failure, common autoimmune diseases (rheumatoid arthritis, systemic lupus erythematosus, or psoriasis), and immunosuppression (HIV infection, sickle cell disease, organ transplant, or asplenia). All codelists are available for review and reuse.^29^

### Statistical analysis

Sociodemographic and clinical characteristics at baseline were summarised using descriptive statistics, stratified by ethnic group. Follow-up began on Feb 1, 2020, for wave 1 and on Sept 1, 2020, for wave 2, and ended at the earliest occurrence of the outcome of interest, death, deregistration from a primary care practice, or the censoring date for the dataset capturing the outcome of interest (from July 30 to Aug 3, 2020, [for wave 1] or to Dec 31, 2020 [for wave 2]).

Multivariable Cox proportional hazards regression was used to estimate ethnic differences in the cause-specific hazard of each outcome in the whole denominator population.^30^ All analyses were adjusted for age (using restricted cubic splines), sex, deprivation quintile, diagnosed comorbidities, BMI, HbA_1c_, blood pressure, number of primary care consultations in the preceding 12 months, and household size. To investigate the extent to which age-sex-adjusted ethnicity-associated differences could further be explained by deprivation, comorbidities, and household size, we sequentially adjusted for age and sex in the first model, adding deprivation in the second model, comorbidities, clinical factors, and primary care consultations in the third model, and household size in the fourth model. All models were stratified by STP to account for clustering by geographical region. All analyses were done separately for wave 1 and wave 2. Analyses of COVID-19-related hospital admissions for wave 1 were added during the revision stage of this Article as data on hospitalisations became available in OpenSAFELY after the initial submission; therefore, cohort sizes slightly differ for this outcome compared with other outcomes.

In secondary analyses, we estimated ethnic differences in the risk of non-COVID-19 death (defined as any death without a COVID-19 diagnosis code anywhere on the death certificate). Additionally, we used logistic regression adjusting for all covariates to examine ethnic differences in the odds of testing positive among those tested for SARS-CoV-2. We also estimated differences between ethnic groups in all outcomes for care home residents, adjusting for all covariates except for household size.

In sensitivity analyses, we used multiple imputation to account for missing ethnicity data, examining differences between ethnic groups in the risk of death where COVID-19 was the underlying cause (rather than any cause) and exploring the effect of regional variation on ethnic differences in all outcomes. Proportional hazards assumptions were assessed by testing for a zero slope in the scaled Schoenfeld residuals and graphical inspection of Kaplan-Meier plots.

Data management was done with Python 3.8 and SQL, and analysis was done using Stata 16.

### Role of the funding source

The funders of the study had no role in study design, data collection, data analysis, data interpretation, or writing of the report.

## Results

**Figure 1:**
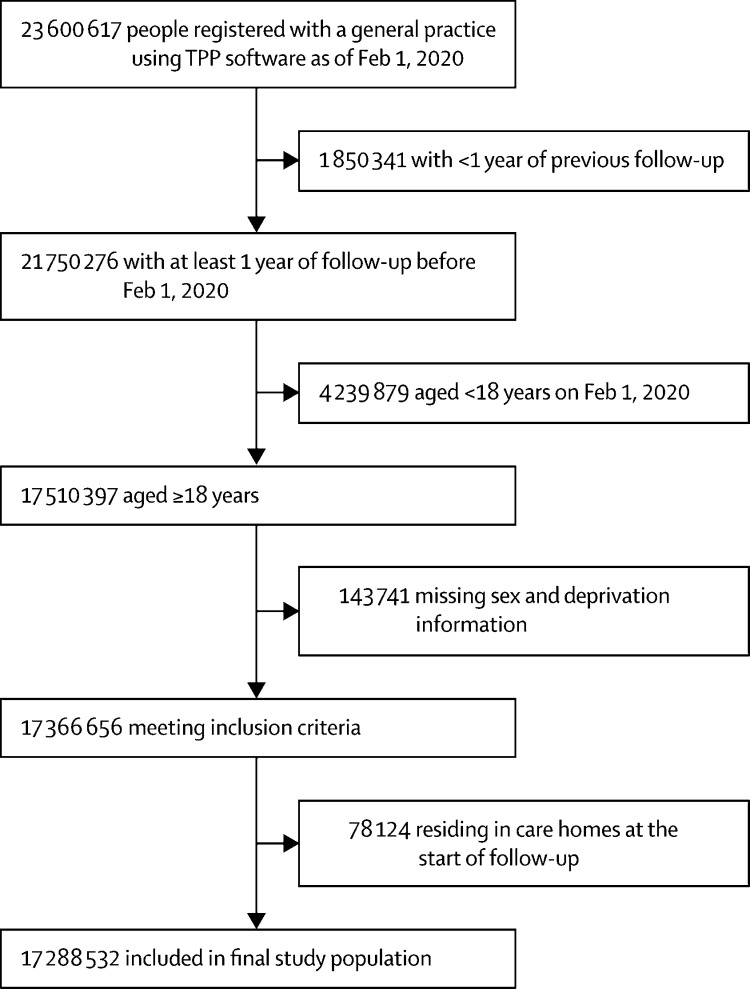
Study population flowchart

From a total of 23 600 617 individuals in the OpenSAFELY database on Feb 1, 2020, 17 288 532 adults aged 18 years or older who met the selection criteria were included in the study (figure 1). The ethnic breakdown of the study population was 10 877 978 (62·9%) people of White ethnicity, 1 025 319 (5·9%) of South Asian ethnicity, 340 912 (2·0%) of Black ethnicity, 320 788 (1·9%) of other ethnicity, 170 484 (1·0%) of mixed ethnicity, and 4 553 051 (26·3%) of unknown ethnicity. Compared with the White population, minority ethnic groups were younger and over-represented in deprived neighbourhoods, large households, and diabetic populations (table 1; appendix p 3).

**Table 1:** Baseline characteristics by ethnic group

		**Overall (n=17 288 532)**	**White (n=10 877 978)**	**South Asian (n=1 025 319)**	**Black (n=340 912)**	**Mixed (n=170 484)**	**Other (n=320 788)**	**Unknown (n=4 553 051)**
Age, years		49·6 (18·6)	51·2 (18·2)	43·0 (15·4)	43·8 (15·5)	40·0 (14·9)	40·2 (15·0)	48·8 (20·0)
Sex								
	Female	8 638 780 (50·0%)	5 650 496 (51·9%)	490 641 (47·9%)	169 832 (49·8%)	87 678 (51·4%)	157 832 (49·2%)	2 082 301 (45·7%)
	Male	8 649 752 (50·0%)	5 227 482 (48·1%)	534 678 (52·1%)	171 080 (50·2%)	82 806 (48·6%)	162 956 (50·8%)	2 470 750 (54·3%)
Index of Multiple Deprivation								
	1 (most affluent)	3 495 006 (20·2%)	2 294 962 (21·1%)	87 798 (8·6%)	22 716 (6·7%)	22 259 (13·1%)	43 209 (13·5%)	1 024 062 (22·5%)
	2	3 481 686 (20·1%)	2 292 018 (21·1%)	114 552 (11·2%)	34 646 (10·2%)	27 255 (16·0%)	54 638 (17·0%)	958 577 (21·1%)
	3	3 487 259 (20·2%)	2 220 520 (20·4%)	189 485 (18·5%)	54 731 (16·1%)	32 256 (18·9%)	60 843 (19·0%)	929 424 (20·4%)
	4	3 482 514 (20·1%)	2 113 382 (19·4%)	295 273 (28·8%)	90 963 (26·7%)	40 543 (23·8%)	79 661 (24·8%)	862 692 (18·9%)
	5 (most deprived)	3 342 067 (19·3%)	1 957 096 (18·0%)	338 211 (33·0%)	137 856 (40·4%)	48 171 (28·3%)	82 437 (25·7%)	778 296 (17·1%)
Number of people in household								
	1–2	7 533 408 (43·6%)	5 169 700 (47·5%)	209 599 (20·4%)	118 164 (34·7%)	62 417 (36·6%)	118 594 (37·0%)	1 854 934 (40·7%)
	3–5	6 166 295 (35·7%)	3 762 179 (34·6%)	430 932 (42·0%)	135 543 (39·8%)	69 564 (40·8%)	117 587 (36·7%)	1 650 490 (36·3%)
	6–10	992 375 (5·7%)	443 780 (4·1%)	221 312 (21·6%)	50 678 (14·9%)	18 183 (10·7%)	39 841 (12·4%)	218 581 (4·8%)
	≥11	169 045 (1·0%)	75 056 (0·7%)	41 039 (4·0%)	6486 (1·9%)	3475 (2·0%)	14 228 (4·4%)	28 761 (0·6%)
	Unknown	2 427 409 (14·0%)	1 427 263 (13·1%)	122 437 (11·9%)	30 041 (8·8%)	16 845 (9·9%)	30 538 (9·5%)	800 285 (17·6%)
Number of general practitioner consultations in the 12 months before baseline		3·0 (0·0–8·0)	4·0 (1·0–8·0)	3·0 (0·0–8·0)	3·0 (0·0–7·0)	2·0 (0·0–7·0)	1·0 (0·0–5·0)	2·0 (0·0–7·0)
Smoking status								
	Never*	8 653 213 (50·1%)	4 882 899 (44·9%)	751 034 (73·2%)	232 984 (68·3%)	96 363 (56·5%)	208 450 (65·0%)	2 481 483 (54·5%)
	Former	5 683 564 (32·9%)	4 037 473 (37·1%)	159 103 (15·5%)	63 290 (18·6%)	39 798 (23·3%)	58 768 (18·3%)	1 325 132 (29·1%)
	Current	2 951 755 (17·1%)	1 957 606 (18·0%)	115 182 (11·2%)	44 638 (13·1%)	34 323 (20·1%)	53 570 (16·7%)	746 436 (16·4%)
BMI, kg/m^2^		27·4 (5·7)	27·5 (5·7)	26·6 (5·1)	28·0 (5·7)	26·8 (5·7)	25·2 (5·1)	27·7 (5·9)
BMI category, with adjustment for South Asian populations								
	Underweight	294 735 (1·7%)	180 046 (1·7%)	26 124 (2·5%)	5012 (1·5%)	3800 (2·2%)	11 844 (3·7%)	67 909 (1·5%)
	Normal	4 571 011 (26·4%)	3 234 076 (29·7%)	176 170 (17·2%)	81 236 (23·8%)	53 668 (31·5%)	120 216 (37·5%)	905 645 (19·9%)
	Overweight	4 666 217 (27·0%)	3 218 869 (29·6%)	313 478 (30·6%)	97 270 (28·5%)	42 901 (25·2%)	71 463 (22·3%)	922 236 (20·3%)
	Obese I	2 457 922 (14·2%)	1 633 030 (15·0%)	212 808 (20·8%)	54 909 (16·1%)	20 662 (12·1%)	27 228 (8·5%)	509 285 (11·2%)
	Obese II	949 367 (5·5%)	631 273 (5·8%)	71 428 (7·0%)	21 383 (6·3%)	7825 (4·6%)	8355 (2·6%)	209 103 (4·6%)
	Obese III	474 090 (2·7%)	318 199 (2·9%)	28 812 (2·8%)	10 282 (3·0%)	4076 (2·4%)	3505 (1·1%)	109 216 (2·4%)
	Unknown	3 875 190 (22·4%)	1 662 485 (15·3%)	196 499 (19·2%)	70 820 (20·8%)	37 552 (22·0%)	78 177 (24·4%)	1 829 657 (40·2%)
HbA_1c_, %		5·9 (1·0)	5·8 (1·0)	6·1 (1·2)	6·0 (1·2)	5·9 (1·1)	5·8 (1·0)	5·9 (1·0)
HbA_1c_, mmol/mol		41·0 (92·7)	40·4 (62·2)	44·3 (218·2)	42·8 (138·9)	40·5 (12·0)	40·2 (10·7)	41·4 (101·9)
HbA_1c_ category								
	<6·5%	6 706 373 (38·8%)	4 546 662 (41·8%)	424 221 (41·4%)	127 134 (37·3%)	54 728 (32·1%)	92 552 (28·9%)	1 461 076 (32·1%)
	6·5–7·4%	582 059 (3·4%)	350 237 (3·2%)	64 385 (6·3%)	15 294 (4·5%)	4500 (2·6%)	7680 (2·4%)	139 963 (3·1%)
	7·5–7·9%	155 580 (0·9%)	94 158 (0·9%)	17 117 (1·7%)	3376 (1·0%)	1144 (0·7%)	1825 (0·6%)	37 960 (0·8%)
	8·0–8·9%	168 963 (1·0%)	103 062 (0·9%)	18 312 (1·8%)	3558 (1·0%)	1226 (0·7%)	1965 (0·6%)	40 840 (0·9%)
	≥9·0%	190 305 (1·1%)	115 910 (1·1%)	21 060 (2·1%)	5415 (1·6%)	1686 (1·0%)	2215 (0·7%)	44 019 (1·0%)
	Unknown	9 485 252 (54·9%)	5 667 949 (52·1%)	480 224 (46·8%)	186 135 (54·6%)	107 200 (62·9%)	214 551 (66·9%)	2 829 193 (62·1%)
Blood pressure (mm Hg)								
	Systolic	128·0 (15·7)	128·3 (15·5)	123·8 (15·4)	127·1 (16·0)	124·0 (15·3)	122·5 (15·4)	129·0 (15·9)
	Diastolic	76·8 (9·9)	76·8 (9·8)	76·5 (9·7)	78·0 (10·3)	76·6 (10·1)	75·8 (10·0)	76·7 (10·0)
Blood pressure category								
	Normal	2 653 823 (15·4%)	1 719 408 (15·8%)	211 095 (20·6%)	56 786 (16·7%)	32 711 (19·2%)	57 475 (17·9%)	576 348 (12·7%)
	Elevated	1 818 544 (10·5%)	1 225 760 (11·3%)	99 291 (9·7%)	32 093 (9·4%)	15 713 (9·2%)	23 598 (7·4%)	422 089 (9·3%)
	High, stage I	4 270 604 (24·7%)	2 880 135 (26·5%)	238 962 (23·3%)	80 605 (23·6%)	35 154 (20·6%)	53 458 (16·7%)	982 290 (21·6%)
	High, stage II	3 010 178 (17·4%)	2 025 395 (18·6%)	120 401 (11·7%)	54 017 (15·8%)	19 008 (11·1%)	26 433 (8·2%)	764 924 (16·8%)
	Unknown	5 535 383 (32·0%)	3 027 280 (27·8%)	355 570 (34·7%)	117 411 (34·4%)	67 898 (39·8%)	159 824 (49·8%)	1 807 400 (39·7%)
Comorbidities								
	Type 1 diabetes	88 294 (0·5%)	59 940 (0·6%)	2508 (0·2%)	1363 (0·4%)	637 (0·4%)	637 (0·2%)	23 209 (0·5%)
	Type 2 diabetes	1 234 858 (7·1%)	747 798 (6·9%)	135 741 (13·2%)	31 590 (9·3%)	9603 (5·6%)	15 527 (4·8%)	294 599 (6·5%)
	Diagnosed hypertension	3 703 816 (21·4%)	2 437 571 (22·4%)	173 237 (16·9%)	69 524 (20·4%)	20 712 (12·1%)	31 706 (9·9%)	971 066 (21·3%)
	Chronic heart disease	1 193 155 (6·9%)	803 572 (7·4%)	53 903 (5·3%)	11 092 (3·3%)	4731 (2·8%)	8136 (2·5%)	311 721 (6·8%)
	Stroke	368 707 (2·1%)	249 742 (2·3%)	12 429 (1·2%)	4373 (1·3%)	1495 (0·9%)	2161 (0·7%)	98 507 (2·2%)
	Chronic kidney disease†	978 300 (5·7%)	647 085 (5·9%)	27 979 (2·7%)	15 042 (4·4%)	3787 (2·2%)	4782 (1·5%)	279 625 (6·1%)
	End-stage renal failure	25 348 (0·1%)	14 615 (0·1%)	2410 (0·2%)	878 (0·3%)	219 (0·1%)	352 (0·1%)	6874 (0·2%)
	Cancer	979 433 (5·7%)	684 824 (6·3%)	19 868 (1·9%)	9249 (2·7%)	3718 (2·2%)	6640 (2·1%)	255 134 (5·6%)
	Autoimmune disease	889 832 (5·1%)	615 132 (5·7%)	37 348 (3·6%)	6912 (2·0%)	4988 (2·9%)	7504 (2·3%)	217 948 (4·8%)
	Immunosuppression	93 162 (0·5%)	54 143 (0·5%)	4602 (0·4%)	10 087 (3·0%)	2118 (1·2%)	1425 (0·4%)	20 787 (0·5%)
	Chronic liver disease	104 781 (0·6%)	69 901 (0·6%)	5677 (0·6%)	3035 (0·9%)	894 (0·5%)	2445 (0·8%)	22 829 (0·5%)
	Dementia	34 169 (0·2%)	22 855 (0·2%)	1199 (0·1%)	538 (0·2%)	106 (0·1%)	195 (0·1%)	9276 (0·2%)
	Neurological disease	169 483 (1·0%)	116 300 (1·1%)	5803 (0·6%)	1951 (0·6%)	888 (0·5%)	1163 (0·4%)	43 378 (1·0%)
	Asthma	2 663 321 (15·4%)	1 790 975 (16·5%)	125 745 (12·3%)	37 351 (11·0%)	25 776 (15·1%)	22 406 (7·0%)	661 068 (14·5%)
	Chronic respiratory disease	718 047 (4·2%)	527 265 (4·8%)	17 435 (1·7%)	5003 (1·5%)	2274 (1·3%)	3520 (1·1%)	162 550 (3·6%)

Data are mean (SD), n (%), or median (IQR). BMI=body-mass index. HbA_1c_=glycated haemoglobin.

* Including those with missing data.

† Defined as estimated glomerular filtration rate <60 mL/min per 1·73 m^2^.

**Figure 2:**
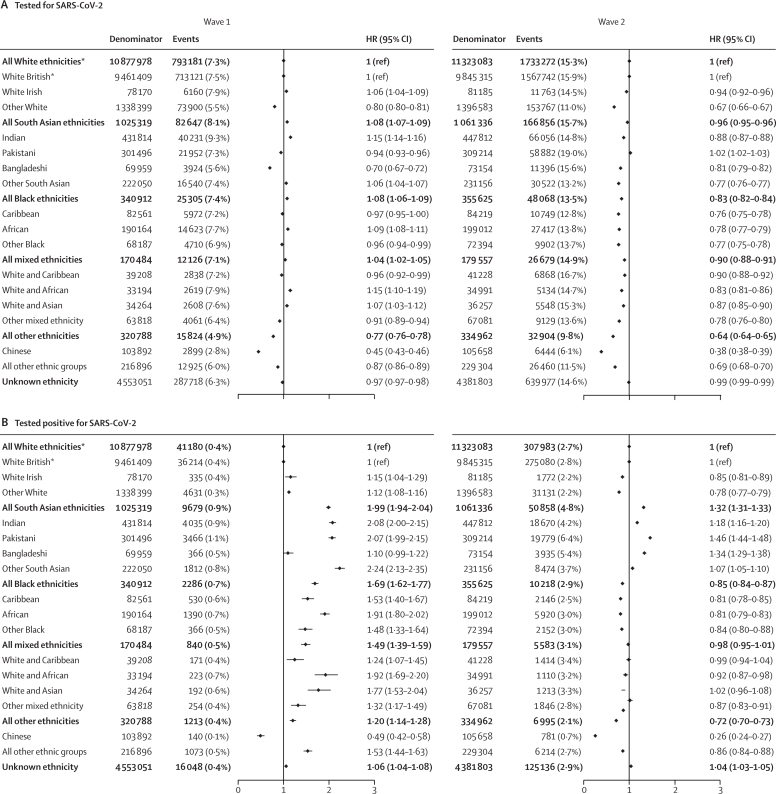
Ethnic differences in the risks of being tested for SARS-CoV-2 infection (A) and testing positive for SARS-CoV-2 infection (B) Models adjust for age, sex, deprivation quintile, all prespecified clinical comorbidities, body-mass index category, glycated haemoglobin category, systolic and diastolic blood pressure category, number of primary care consultations in the previous 12 months, household size, and stratification by sustainability and transformation partnership region. HR=hazard ratio. *All White ethnicities is the reference category for comparison of ethnicity in the five broad categories, and White British is the reference category for comparison of ethnicity in the 16 subcategories.

Between Feb 1 and Aug 3, 2020 (wave 1), 1 216 801 (7·0%) people in the study population received a test for SARS-CoV-2 infection, and 71 246 (0·4%) tested positive (table 2). The ethnic breakdown of individuals who received a test was similar to that of the general population, although test recipients were slightly older with more comorbid chronic conditions than the general population (appendix p 4). After accounting for all measured explanatory variables (age, sex, deprivation, comorbidities, clinical factors, primary care consultations in the preceding 12 months, and household size, with stratification by STP region), South Asian, Black, and mixed ethnicity groups were more likely to be tested (South Asian group adjusted hazard ratio [HR] 1·08 [95% CI 1·07–1·09], Black group 1·08 [1·06–1·09], mixed ethnicity group 1·04 [1·02–1·05]) and to test positive for SARS-CoV-2 (1·99 [1·94–2·04], 1·69 [1·62–1·77], 1·49 [1·39–1·59]) compared with the White ethnic group (figure 2). Across the 16 subcategories of ethnicity, risks of testing positive were similar to those of the respective high-level categories, except for the Chinese group, for whom risks of being tested and testing positive (0·49 [0·42–0·58]) were lower than for the White British group. When restricted to the population who had ever received a test (as opposed to the whole denominator population used in the primary analysis), patterns by ethnic group remained unchanged, except for the Chinese group, who had an equivalent risk of testing positive (odds ratio [OR] 1·13 [95% CI 0·95–1·34], adjusted for all explanatory variables; appendix p 15).

**Table 2:** Associations between ethnicity in five categories and COVID-19 outcomes in wave 1, with serial adjustment

	**Denominator**	**Events**	**Hazard ratio (95% CI)**				
			Crude	Age-sex adjusted	Plus deprivation	Plus comorbidities and clinical factors*	Plus household size
**Tested for SARS-CoV-2 (n=1 216 801)**							
White	10 877 978	793 181 (7·3%)	1 (ref)	1 (ref)	1 (ref)	1 (ref)	1 (ref)
South Asian	1 025 319	82 647 (8·1%)	1·08 (1·08–1·09)	1·11 (1·10–1·12)	1·09 (1·08–1·09)	1·09 (1·08–1·09)	1·08 (1·07–1·09)
Black	340 912	25 305 (7·4%)	1·04 (1·03–1·05)	1·08 (1·06–1·09)	1·04 (1·03–1·06)	1·08 (1·06–1·09)	1·08 (1·06–1·09)
Mixed	170 484	12 126 (7·1%)	1·00 (0·98–1·01)	1·03 (1·01–1·05)	1·01 (1·00–1·03)	1·04 (1·02–1·06)	1·04 (1·02–1·05)
Other	320 788	15 824 (4·9%)	0·68 (0·67–0·69)	0·71 (0·69–0·72)	0·70 (0·69–0·71)	0·78 (0·77–0·79)	0·77 (0·76–0·78)
Unknown	4 553 051	287 718 (6·3%)	0·86 (0·86–0·87)	0·88 (0·88–0·89)	0·89 (0·88–0·89)	0·97 (0·97–0·98)	0·97 (0·97–0·98)
**Tested positive for SARS-CoV-2 (n=71 246)**							
White	10 877 978	41 180 (0·4%)	1 (ref)	1 (ref)	1 (ref)	1 (ref)	1 (ref)
South Asian	1 025 319	9679 (0·9%)	2·38 (2·32–2·43)	2·64 (2·57–2·70)	2·45 (2·39–2·51)	2·16 (2·10–2·21)	1·99 (1·94–2·04)
Black	340 912	2286 (0·7%)	1·82 (1·74–1·90)	2·04 (1·95–2·13)	1·86 (1·78–1·94)	1·74 (1·67–1·82)	1·69 (1·62–1·77)
Mixed	170 484	840 (0·5%)	1·37 (1·28–1·46)	1·59 (1·48–1·70)	1·52 (1·42–1·63)	1·51 (1·41–1·62)	1·49 (1·39–1·59)
Other	320 788	1213 (0·4%)	1·06 (1·00–1·12)	1·22 (1·15–1·29)	1·18 (1·11–1·25)	1·25 (1·18–1·33)	1·20 (1·14–1·28)
Unknown	4 553 051	16 048 (0·4%)	0·97 (0·95–0·99)	0·99 (0·98–1·01)	1·00 (0·98–1·02)	1·06 (1·04–1·08)	1·06 (1·04–1·08)
**COVID-19-related hospital admission**†**(n=32 473)**							
White	11 110 312	20 504 (0·2%)	1 (ref)	1 (ref)	1 (ref)	1 (ref)	1 (ref)
South Asian	1 026 551	2836 (0·3%)	1·26 (1·21–1·31)	2·04 (1·96–2·13)	1·83 (1·76–1·91)	1·59 (1·52–1·66)	1·48 (1·41–1·55)
Black	342 561	1051 (0·3%)	1·39 (1·30–1·48)	2·18 (2·05–2·33)	1·89 (1·77–2·01)	1·81 (1·70–1·94)	1·78 (1·67–1·90)
Mixed	171 933	302 (0·2%)	0·86 (0·77–0·97)	1·78 (1·59–1·99)	1·64 (1·47–1·84)	1·65 (1·47–1·85)	1·63 (1·45–1·83)
Other	323 529	504 (0·2%)	0·77 (0·71–0·85)	1·52 (1·39–1·66)	1·43 (1·31–1·57)	1·59 (1·45–1·74)	1·54 (1·41–1·69)
Unknown	4 408 386	7276 (0·2%)	0·91 (0·88–0·93)	0·96 (0·94–0·99)	0·97 (0·94–0·99)	1·06 (1·03–1·09)	1·06 (1·03–1·09)
**COVID-19-related intensive care unit admission (n=3096)**							
White‡	10 877 978	1700 (<0·1%)	1 (ref)	1 (ref)	1 (ref)	1 (ref)	1 (ref)
South Asian	1 025 319	410 (<0·1%)	2·38 (2·12–2·67)	3·30 (2·93–3·71)	3·05 (2·71–3·44)	2·34 (2·07–2·64)	2·18 (1·92–2·48)
Black	340 912	186 (0·1%)	3·08 (2·63–3·60)	3·91 (3·34–4·58)	3·52 (3·00–4·13)	3·21 (2·73–3·77)	3·12 (2·65–3·67)
Mixed	170 484	56 (<0·1%)	1·97 (1·51–2·57)	3·19 (2·44–4·17)	3·01 (2·30–3·94)	3·02 (2·31–3·95)	2·96 (2·26–3·87)
Other	320 788	97 (<0·1%)	1·83 (1·48–2·25)	2·92 (2·37–3·60)	2·80 (2·27–3·45)	3·28 (2·66–4·06)	3·18 (2·58–3·93)
Unknown	4 553 051	662 (<0·1%)	0·95 (0·87–1·04)	1·03 (0·94–1·12)	1·03 (0·94–1·13)	1·09 (0·99–1·19)	1·08 (0·99–1·19)
**COVID-19-related death (n=11 649)**							
White	10 877 978	7514 (0·1%)	1 (ref)	1 (ref)	1 (ref)	1 (ref)	1 (ref)
South Asian	1 025 319	734 (0·1%)	0·89 (0·83–0·97)	1·85 (1·71–2·01)	1·64 (1·52–1·78)	1·47 (1·35–1·60)	1·26 (1·15–1·37)
Black	340 912	268 (0·1%)	0·96 (0·85–1·09)	1·88 (1·66–2·13)	1·61 (1·42–1·82)	1·50 (1·32–1·70)	1·51 (1·33–1·71)
Mixed	170 484	65 (<0·1%)	0·51 (0·40–0·65)	1·58 (1·23–2·01)	1·44 (1·13–1·84)	1·43 (1·12–1·83)	1·41 (1·11–1·81)
Other	320 788	107 (<0·1%)	0·44 (0·37–0·54)	1·25 (1·03–1·51)	1·17 (0·97–1·42)	1·24 (1·02–1·50)	1·22 (1·00–1·48)
Unknown	4 553 051	2961 (0·1%)	0·95 (0·91–0·99)	0·92 (0·88–0·96)	0·93 (0·89–0·97)	1·00 (0·95–1·04)	1·01 (0·97–1·06)

Data are N, n (%), or hazard ratio (95% CI).

* Including number of primary care consultations in the preceding 12 months.

† Analyses of COVID-19-related hospital admissions for wave 1 were added during the revision stage of this Article as data on hospitalisations became available in OpenSAFELY after the initial submission; therefore, cohort sizes differ for this outcome compared with other outcomes.

‡ For categories containing small numbers (≤5) within any subcategory, we have rounded all counts to the nearest 10, per data disclosure agreements.

**Figure 3:**
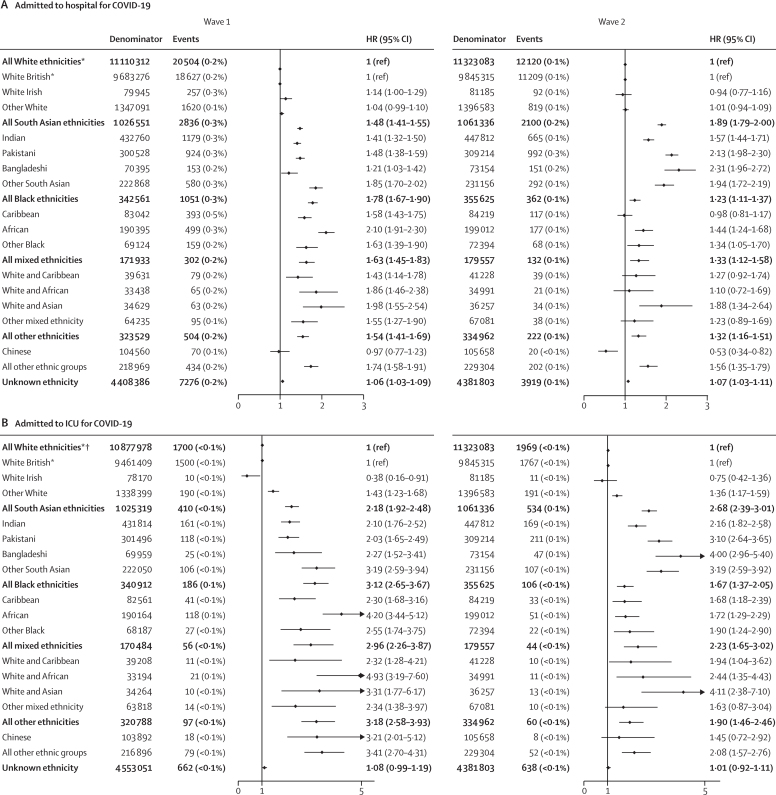
Ethnic differences in the risk of COVID-19-related hospital admission (A) and COVID-19-related ICU admission (B) Models adjust for age, sex, deprivation quintile, all prespecified clinical comorbidities, body-mass index category, glycated haemoglobin category, systolic and diastolic blood pressure category, number of primary care consultations in the previous 12 months, household size, and stratification by sustainability and transformation partnership region. Analyses of COVID-19-related hospital admissions for wave 1 were added during the revision stage of this Article as data on hospitalisations became available in OpenSAFELY after the initial submission; therefore, cohort sizes differ for this outcome compared with other outcomes. HR=hazard ratio. ICU=intensive care unit. *All White ethnicities is the reference category for comparison of ethnicity in the five broad categories, and White British is the reference category for comparison of ethnicity in the 16 subcategories. †For categories containing small numbers (≤5) within any subcategory, we have rounded all counts to the nearest 10, per data disclosure agreements.

**Figure 4:**
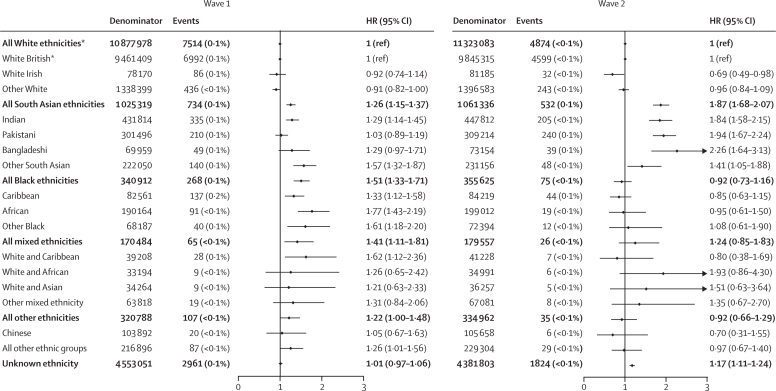
Ethnic differences in the risk of COVID-19-related death Models adjust for age, sex, deprivation quintile, all prespecified clinical comorbidities, body-mass index category, glycated haemoglobin category, systolic and diastolic blood pressure category, number of primary care consultations in the previous 12 months, household size, and stratification by sustainability and transformation partnership region. For categories containing small numbers (≤5) within any subcategory, we have rounded all counts to the nearest 10, per data disclosure agreements. HR=hazard ratio. *All White ethnicities is the reference category for comparison of ethnicity in the five broad categories, and White British is the reference category for comparison of ethnicity in the 16 subcategories.

Between Feb 1 and Aug 3, 2020, 32 473 (0·2%) individuals in the study population were admitted to hospital for COVID-19, 3096 (<0·1%) were admitted to ICU for COVID-19, and there were 11 649 (0·1%) COVID-19-related deaths (table 2). After accounting for all measured explanatory factors, risk of hospitalisation was increased, relative to the White reference groups, in all minority ethnic groups (South Asian group adjusted HR 1·48 [95% CI 1·41–1·55], Black group 1·78 [1·67–1·90], mixed ethnicity group 1·63 [1·45–1·83], other ethnicity group 1·54 [1·41–1·69]), including all minority ethnic subcategories except the Chinese group (0·97 [0·77–1·23]; figure 3A). Risk of ICU admission was around 2–3 times in the four broad minority ethnic groups (South Asian group 2·18 [1·92–2·48], Black group 3·12 [2·65–3·67], mixed ethnicity group 2·96 [2·26–3·87], other ethnicity group 3·18 [2·58–3·93]) relative to the White reference group, and around 2–5 times higher among South Asian, Black, mixed, and other ethnic subcategories relative to the White British group (figure 3B). Risk of COVID-19-related death was increased by 22–51% in the four broad minority ethnic groups relative to the White group (South Asian group 1·26 [1·15–1·37], Black group 1·51 [1·31–1·71], mixed ethnicity group 1·41 [1·11–1·81], other ethnicity group 1·22 [1·00–1·48]; figure 4).

After accounting for age and sex, further adjustment had little effect on the likelihood of being tested for COVID-19. In South Asian groups, adjustment for clinical characteristics led to the largest reduction in HRs for testing positive for SARS-CoV-2 and hospitalisation and ICU admission due to COVID-19, and adjustment for deprivation and household size made equivalent reductions in HRs for COVID-19-related mortality. In all other minority ethnic groups, adjustment for social deprivation led to the largest reduction in HRs for all outcomes after accounting for age and sex (table 2; appendix p 5).

Between Sept 1 and Dec 31, 2020 (wave 2), 2 647 756 (15·3%) individuals in the study population received a test for SARS-CoV-2, 506 773 (2·9%) tested positive, 18 885 (0·1%) were admitted to hospital for COVID-19, 3351 (<0·1%) had a COVID-19 related ICU admission, and there were 7366 (<0·1%) COVID-19-related deaths. In contrast to wave 1, all four of the broad minority ethnic groups were less likely to be tested than the White group, and this pattern was also seen in all minority ethnic subcategories relative to the White British subcategory, with the exception of the Pakistani group (figure 2). The South Asian group (and each of its subgroups) remained at higher risk of testing positive (adjusted HR 1·32 [95% CI 1·31–1·33]) than the White group, and the risks of COVID-19-related hospitalisation (1·89 [1·79–2·00]), ICU admission (2·68 [2·39–3·01]), and death (1·87 [1·68–2·07]) were relatively greater in magnitude in wave 2 than in wave 1 (Figure 2, Figure 3, Figure 4). In contrast to wave 1, the Black group (and all Black subgroups) was less likely than the White group to test positive (0·85 [0·84–0·87]; figure 2), although the risk of testing positive remained higher among those ever tested (OR 1·03 [95% CI 1·02–1·06] adjusted for all explanatory variables; appendix p 15), rather than the general denominator population. Risks of COVID-19-related hospitalisation (adjusted HR 1·23 [95% CI 1·11–1·37]) and ICU admission (1·67 [1·37–2·05]) remained higher for the Black group than for the White group in wave 2, but these risks were attenuated in magnitude compared with wave 1 (figure 3). However, in contrast to the pattern seen in the broader Black group, the risk of COVID-19-related hospitalisation in the Caribbean subgroup did not differ from that of the White British group (0·98 [0·81–1·17]). Additionally, the excess risk of COVID-19 death was attenuated for the Black group in wave 2 compared with wave 1, so that no differences between the White and Black groups remained in wave 2 (0·92 [0·73–1·16]; figure 4).

71 920 non-COVID-19-related deaths occurred in the study population during wave 1. The risk of non-COVID-19-related death was 15–32% lower in all non-White ethnic groups (South Asian group adjusted HR 0·85 [95% CI 0·81–0·90], Black group 0·85 [0·78–0·92], mixed ethnicity group 0·81 [0·70–0·93]; other ethnicity group 0·68 [0·61–0·77]) than in the White group after adjustment for all measured explanatory variables (appendix p 5). In wave 2, the risk of non-COVID-19-related death remained lower for the South Asian, Black, and other ethnicity groups than for the White group (appendix p 10).

In wave 1, among the 78 124 care home residents, 46 065 (59·0%) individuals were tested for SARS-CoV-2, 6330 (8·1%) tested positive, 2517 (3·2%) were admitted to hospital, and 3877 (5·0%) died from COVID-19. Although no ethnic differences in being tested for or testing positive for SARS-CoV-2 infection were apparent, people of Black ethnicity (adjusted HR 1·43 [95% CI 1·02–2·00]) and other ethnicity (1·73 [1·19–2·50]) were more likely to die from COVID-19 than people of White ethnicity, after adjustment for all measured explanatory variables except household size. In wave 2, no ethnic differences among care home populations were evident for any outcome except COVID-19 death, which was raised for South Asian groups (1·81 [1·07–3·05]; appendix p 16). Because of small numbers, we were unable to explore ethnic differences in ICU admissions or differences according to ethnicity in 16 categories among care home residents.

Using multiple imputation to account for unknown ethnicity did not materially change any of the associations observed in the complete case analysis (appendix p 17), nor did restricting the definition of COVID-19-related death to underlying cause only (appendix p 18) or removing adjustment for STP region (appendix p 19). We detected no evidence of deviations from the proportional hazards assumption (appendix p 20).

## Discussion

In a population-based cohort study of 17 million adults in England we found that, although ethnic differences in testing were small, minority ethnic groups were at increased risk of testing positive for SARS-CoV-2 and of COVID-19-related hospitalisation, ICU admission, and death. Disaggregation into detailed ethnic categories revealed important within-group heterogeneity, emphasising the importance of disaggregated reporting wherever possible. In wave 2, minority ethnic groups were less likely to be tested than White groups, and risks of severe COVID-19 outcomes (ie, hospitalisation, ICU admission, and death) increased for South Asian groups but were attenuated in all other ethnic groups relative to the White group compared with wave 1.

In the largest UK-based study to date, we captured high-quality clinical data across a range of health-care settings and linked individual-level COVID-19 datasets, which enabled us to generate timely insights into ethnic disparities at different severity levels of COVID-19, from being tested for infection to dying from the disease. We were able to report findings according to self-reported ethnicity in 16 categories, whereas other UK-based studies have aggregated ethnicity into higher-level groups because of small numbers. We also reported differences in outcomes using a general population-based sample, which allowed us to overcome issues commonly faced by studies limited to individuals with SARS-CoV-2 infection or hospitalisation, wherein the populations under study might not represent the true general population at risk.^31^

Our inability to capture all potential explanatory factors of ethnic disparities in COVID-19 outcomes is likely to have affected our observed associations. For example, we were unable to account for ethnic differences in genetic ancestry,^32^, ^33^ occupation,^34^ experiences of racism or structural discrimination,^9^, ^35^, ^36^ and health-related behaviour.^37^, ^38^ Because of invalid address information, we were unable to estimate household size for 13% of our population. We might have underestimated household size for homes including people registered at non-TPP primary care practices and overestimated it for individuals living in large apartment blocks, or for people who had not updated their address after moving homes. In recognition of these limitations, we grouped household size into four levels rather than considering it as a continuous measure. Furthermore, it is possible that cause of death might have been misclassified on death certificates, and that the extent of this misclassification might have differed by time period and ethnicity. A limitation of SARS-CoV-2 testing data was the selective opportunity to be tested early in the pandemic, which was skewed towards health-care workers and people with severe or symptomatic disease, particularly during the first wave. Although OpenSAFELY is broadly representative of the English population, it includes data from a single software system that is known to have lower coverage in London than in other regions of the UK. However, our results mirror other studies done in the UK^1^ and in the USA,^5^, ^39^ suggesting that potential mechanisms underpinning ethnic differences in COVID-19 might be common across countries with similar population structures. OpenSAFELY data are collected prospectively in real time by clinicians and practice staff, and are subject to the same strengths and biases as other UK-based electronic health record databases.

Despite these limitations, this study represents the most comprehensive examination of ethnic inequalities in England during the COVID-19 pandemic in 2020. Using the OpenSAFELY data analytics platform, we capitalised on the rapid real-time linkage of routine datasets in a highly secure environment to explore a range of urgent questions around the patterning of ethnic inequalities in the UK.

This study builds on previous research in several ways. First, we have confirmed ethnic differences in COVID-19-related mortality and provided novel data across a range of other outcomes (testing, hospitalisation, and ICU admission). Second, we have explored whether household size has an effect beyond sociodemographic and clinical characteristics. Finally, we have reported on both the general population and care home residents during the first and second waves of the pandemic in England.

Although some minority ethnic groups were less likely to be tested for SARS-CoV-2 in this study, all non-White groups were more likely to test positive, even when restricted to those ever tested. This finding might suggest that White populations are tested more frequently with mild or asymptomatic disease, or that minority ethnic groups get tested at more severe stages of the disease. Disparities in testing might relate to a lack of access to testing sites, poorer health literacy, lack of tailored and accessible health communications, or differences in testing-related behaviours.^40^ Emerging evidence suggests that individuals might avoid seeking a test for fear of losing income or employment if required to quarantine after testing positive.^41^ Given that minority ethnic groups are more likely to work in insecure jobs with poor workplace protections, and in essential or key-worker roles associated with higher risk of death from COVID-19,^42^, ^43^, ^44^ it is likely that social and economic barriers to testing are greater in minority ethnic groups.

Our finding that minority ethnic groups have higher risks of COVID-19-related hospitalisation, ICU admission, and death after accounting for clinical comorbidities suggests that improving equity in clinical care and understanding potential interactions between COVID-19 and underlying conditions are essential for mitigating inequalities in the downstream effects of SARS-CoV-2 infection. The fact that inequalities worsened for South Asian groups in wave 2 compared with in wave 1 suggests that more aggressive and tailored interventions are needed to meet the needs in these communities.^45^ However, our finding of relatively attenuated risks in all other minority ethnic groups is a potential positive finding; further investigation is warranted into which public health actions were most influential in mitigating health disparities for these groups.

Our finding that the magnitude of ethnic differences in testing positive in wave 1 were similar to those in COVID-19-related death suggests that ethnic differences in death might be mediated through exposure or susceptibility to infection, rather than through susceptibility to severe disease once infected. This hypothesis is supported by recent findings from the REACT-2 study, which found higher levels of SARS-CoV-2 antibodies in minority ethnic groups but no ethnic differences in the infection-to-mortality ratio.^46^

After accounting for sociodemographic and clinical factors, household size further explained differences in COVID-19 outcomes for South Asian groups. This finding is consistent with an ONS study that found that multigenerational living was causally associated with an increased risk of death due to COVID-19 in South Asian women, but not in any other ethnic groups.^47^ According to data from the 2011 census, 21% of South Asian groups live in multigenerational households, in contrast to around 7% of White groups.^24^, ^48^ We hypothesise that household size and deprivation might proxy viral exposure by capturing aspects of occupational and community-level exposure. Although multigenerational living might increase the risks of exposure and transmission (from children or working-age adults to older or vulnerable family members), such households and extended communities also offer valuable informal care networks and facilitate engagement with health and community services.^49^ In light of emerging evidence that minority ethnic groups are less likely to take up the COVID-19 vaccine, co-designing culturally competent and non-stigmatising engagement strategies with these communities is increasingly important.^50^, ^51^

National data from England and Scotland have shown that some minority ethnic groups have both better overall health and lower all-cause mortality than White groups.^52^, ^53^ We were able to confirm this pattern in our sensitivity analyses, and our findings of disparities in SARS-CoV-2 positivity and COVID-19-related outcomes, some of which have continued to widen over the course of the epidemic in the UK, are, therefore, particularly concerning.

Our findings mirror large studies in the USA, which have found that minority racial and ethnic communities have elevated risks of testing positive for SARS-CoV-2 infection and of COVID-19-related hospitalisation and death that differentially vary over time, even after accounting for sociodemographic characteristics and underlying health conditions.^5^, ^39^ These parallel findings suggest that mechanisms underpinning ethnic differences in COVID-19 outcomes in England might be common in other settings, and that learnings across settings should be shared.

Improving the quality and completeness of ethnicity data across health and administrative datasets is essential for building a complete picture of ethnic disparities.^54^ Furthermore, although the recording of ethnicity on death certificates has been the norm in Scotland for the past decade, it is only now being considered for use in England.^55^, ^56^, ^57^ Prioritising linkage between health, social, and employment data will be essential in building a complete picture of ethnic differences in COVID-19 risk and outcomes.

Minority ethnic groups in the UK have had disproportionately high levels of poor COVID-19 outcomes, with disparities increasing even within the course of the epidemic for some groups. Reducing ethnic inequalities will need action across a broad range of measures such as addressing the wider adverse effects of disadvantage and structural discrimination, reducing within-household and between-household transmission, and improving control of clinical conditions. The relative importance of each of these measures will differ by both ethnic group and stage of COVID-19 progression. Equality is difficult to achieve, but structural and persistent inequalities must be addressed in a civilised society.

**This online publication has been corrected. The corrected version first appeared at thelancet.com on May 6, 2021**

## Data sharing

All data were linked, stored, and analysed securely within the OpenSAFELY platform. Detailed pseudonymised patient data are potentially reidentifiable and therefore not shared. We rapidly delivered the OpenSAFELY data analysis platform without previous funding to deliver timely analyses of urgent research questions in the context of the global COVID-19 health emergency: now that the platform is established, we are developing a formal process for external users to request access in collaboration with NHS England. Details of this process will be published in the near future on the OpenSAFELY website (https://opensafely.org/).

## Declaration of interests

BG has received research funding from Health Data Research UK, the Laura and John Arnold Foundation, the Wellcome Trust, the National Institute for Health Research (NIHR) Oxford Biomedical Research Centre, the NHS NIHR School of Primary Care Research, the Mohn-Westlake Foundation, the Good Thinking Foundation, the Health Foundation, and WHO; and receives personal income from speaking and writing for lay audiences on the misuse of science. IJD has received unrestricted research grants from and holds shares in GlaxoSmithKline. KK is the director for the University of Leicester Centre for BME Health, Trustee of the South Asian Health Foundation, the NIHR Applied Research Collaboration lead for Ethnicity and Diversity, and a member of the Independent Scientific Advisory Group for Emergencies (SAGE) and chair for the SAGE Ethnicity Subgroup. RM, BG, and RME are members of the SAGE Ethnicity Subgroup. RM reports personal fees from AMGEN. AS is employed by the London School of Hygiene & Tropical Medicine (LSHTM) on a fellowship sponsored by GlaxoSmithKline. All other authors declare no competing interests.
