# Department of Error

**DOI:** 10.1016/S0140-6736(21)01066-7

**Published:** 2021-06-12

*Mathur R, Rentsch CT, Morton CE, et al. Ethnic differences in SARS-CoV-2 infection and COVID-19-related hospitalisation, intensive care unit admission, and death in 17 million adults in England: an observational cohort study using the OpenSAFELY platform.* Lancet *2021;* 397: *1711–24*—In figure 4 of this Article, the written hazard ratios and 95% CIs were incorrect and have been amended. These corrections have been made to the online version as of May 6, 2021, and the printed version is correct.

