# Supplementary material for: Ethnic differences in SARS-CoV-2 infection and COVID-19-related hospitalisation, intensive care unit admission, and death in 17 million adults in England: an observational cohort study using the OpenSAFELY platform
Source: Lancet. 2021 May 8;397(10286):1711–24. doi: 10.1016/S0140-6736(21)00634-6 (PMC8087292; doi:10.1016/S0140-6736(21)00634-6)
Supplement: Supplementary appendix [file mmc1.pdf]

THE LANCET

Supplementary appendix

This appendix formed part of the original submission and has been peer reviewed. We post it as supplied by the authors.

Supplement to: Mathur R, Rentsch CT, Morton CE, et al. Ethnic differences in SARS-CoV-2 infection and COVID-19-related hospitalisation, intensive care unit admission, and death in 17 million adults in England: an observational cohort study using the OpenSAFELY platform. *Lancet* 2021; published online April 30. http://dx.doi.org/10.1016/S0140-6736(21)00634-6.

SUPPLEMENTARY APPENDIX ....

OPEN SOURCE MATERIALS: 2

INFORMATION GOVERNANCE AND ETHICS 2

PATIENT AND PUBLIC INVOLVEMENT 2

TABLE S1. BASELINE CHARACTERISTICS BY ETHNIC GROUP IN 16 CATEGORIES 3

TABLE S2. COMPARISON OF CHARACTERISTICS OF THE GENERAL POPULATION VS. THOSE RECEIVING A TEST FOR SARS-COV-2 IN WAVES 1 AND 2 4

TABLE S3. ASSOCIATION BETWEEN ETHNICITY IN 5 CATEGORIES AND NON-COVID-19 RELATED MORTALITY WAVE 1 (SERIAL ADJUSTMENT) 5

TABLE S4. ASSOCIATION BETWEEN ETHNICITY IN 16 CATEGORIES AND COVID-19 OUTCOMES IN WAVE 1 (SERIAL ADJUSTMENT) 5

TABLE S5. ASSOCIATION BETWEEN ETHNICITY IN 5 CATEGORIES AND COVID-19 OUTCOMES IN WAVE 2 (SERIAL ADJUSTMENT) 9

TABLE S6. ASSOCIATION BETWEEN ETHNICITY IN 16 CATEGORIES AND COVID-19 OUTCOMES IN WAVE 2 (SERIAL ADJUSTMENT) 11

TABLE S7. TESTING THE PROPORTIONAL HAZARDS ASSUMPTIONS FOR EACH OUTCOME (WAVE 1) 14

FIGURE S1. ODDS OF TESTING POSITIVE FOR SARS-COV-2 AMONGST THOSE EVER RECEIVING A TEST 15

FIGURE S2. ASSOCIATION BETWEEN ETHNICITY IN 5 CATEGORIES AND COVID-19 OUTCOMES IN THE CARE-HOME POPULATION IN WAVE 1 AND WAVE 2 16

FIGURE S3. ETHNIC DIFFERENCES IN ALL STUDY OUTCOMES IN WAVE 1: COMPLETE CASE ANALYSIS (CC) VS. MULTIPLE IMPUTATION (MI) IN WAVE 1 17

FIGURE S4. COMPARISON BETWEEN COVID-19 AS ANY CAUSE OF DEATH VS. UNDERLYING CAUSE OF DEATH IN WAVE 1 18

FIGURE S5. ETHNIC DIFFERENCES IN COVID-19 OUTCOMES WITH AND WITHOUT CLUSTERING BY STP REGION IN WAVE 1 19

FIGURE S6. KAPLAN MEIER PLOTS FOR SURVIVAL BY ETHNIC GROUP AND OUTCOME IN WAVE 1 20

DATA ACCESS AND VERIFICATION 21

Open source materials:

Open source materials: All code for data management and analyses and raw outputs are openly shared online for review and re-use (https://github.com/opensafely/ethnicity-covid-research). All iterations of the pre-specified study protocol are archived with version control (https://github.com/opensafely/ethnicity-covid-research/tree/master/protocol). Detailed information on all code lists is openly shared at https://codelists.opensafely.org/ for inspection and reuse.

Information governance and ethics

NHS England is the data controller; TPP is the data processor; and the key researchers on OpenSAFELY are acting on behalf of NHS England. OpenSAFELY is hosted within the TPP environment which is accredited to the ISO 27001 information security standard and is NHS IG Toolkit compliant;1,2 patient data are pseudonymised for analysis and linkage using industry standard cryptographic hashing techniques; all pseudonymised datasets transmitted for linkage onto OpenSAFELY are encrypted; access to the platform is via a virtual private network (VPN) connection, restricted to a small group of researchers who hold contracts with NHS England and only access the platform to initiate database queries and statistical models. The platform includes pseudonymized data such as coded diagnoses, medications and physiological parameters. No free text data are included. This, in addition to other technical and organisational controls, minimizes any risk of re-identification. Similarly pseudonymized datasets from other data providers are securely provided to the EHR vendor and linked to the primary care data.

All database activity is logged; only aggregate statistical outputs leave the platform environment following best practice for anonymization of results such as statistical disclosure control for low cell counts.3 The OpenSAFELY platform adheres to the data protection principles of the UK Data Protection Act 2018 and the EU General Data Protection Regulation (GDPR) 2016. In March 2020, the Secretary of State for Health and Social Care used powers under the UK Health Service (Control of Patient Information) Regulations 2002 (COPI) to require organisations to process confidential patient information for the purposes of protecting public health, providing healthcare services to the public and monitoring and managing the COVID-19 outbreak and incidents of exposure.4 Taken together, these provide the legal bases to link patient datasets on the OpenSAFELY platform. This study was approved by the Health Research Authority (REC reference 20/LO/0651) and by the LSHTM Ethics Board (ref 21863).

Patient and public involvement

Patients were not formally involved in developing this specific study design that was developed rapidly in the context of a global health emergency. We have developed a publicly available website https://opensafely.org/ through which we invite any patient or member of the public to contact us regarding this study or the broader OpenSAFELY project.

Table S1. Baseline characteristics by ethnic group in 16 categories

	British	Irish	Other White	White + Caribbean	White + African	White + Asian	Other mixed	Indian	Pakistani	Bangladeshi	Other Asian	Caribbean	African	Other Black	Chinese	Other
**Denominator**	**9461409**	**78170**	**1338399**	**39208**	**33194**	**34264**	**63818**	**431814**	**301496**	**69959**	**222050**	**82561**	**190164**	**68187**	**103892**	**216896**
Age (years), Mean (SD)	52.3 (18.2)	53.7 (18.5)	42.9 (15.7)	40.8 (16.2)	40.9 (14.1)	39.3 (14.7)	39.4 (14.4)	44.9 (15.9)	41.4 (15.2)	39.9 (14.0)	42.3 (14.8)	50.8 (17.8)	41.2 (13.7)	42.4 (14.8)	37.7 (15.2)	41.4 (14.7)
**Sex**																
Female	4929634 (52.1)	39,401 (50.4)	681,461 (50.9)	21,204 (54.1)	16,468 (49.6)	17,554 (51.2)	32,452 (50.9)	203,092 (47.0)	145,011 (48.1)	32,808 (46.9)	109,730 (49.4)	43,219 (52.3)	92,578 (48.7)	34,035 (49.9)	54,265 (52.2)	103,567 (47.7)
Male	4531775 (47.9)	38,769 (49.6)	656,938 (49.1)	18,004 (45.9)	16,726 (50.4)	16,710 (48.8)	31,366 (49.1)	228,722 (53.0)	156,485 (51.9)	37,151 (53.1)	112,320 (50.6)	39,342 (47.7)	97,586 (51.3)	34,152 (50.1)	49,627 (47.8)	113,329 (52.3)
**Index of Multiple Deprivation (IMD)**																
1 (most affluent)	2110205 (22.3)	14,498 (18.5)	170,259 (12.7)	4,098 (10.5)	3,538 (10.7)	5,946 (17.4)	8,677 (13.6)	49,069 (11.4)	12,084 (4.0)	3,185 (4.6)	23,460 (10.6)	5,105 (6.2)	12,549 (6.6)	5,062 (7.4)	19,824 (19.1)	23,385 (10.8)
2	2036519 (21.5)	16,707 (21.4)	238,792 (17.8)	5,232 (13.3)	4,664 (14.1)	6,501 (19.0)	10,858 (17.0)	58,477 (13.5)	18,848 (6.3)	4,989 (7.1)	32,238 (14.5)	8,419 (10.2)	18,885 (9.9)	7,342 (10.8)	22,253 (21.4)	32,385 (14.9)
3	1933434 (20.4)	16,868 (21.6)	270,218 (20.2)	7,116 (18.1)	5,937 (17.9)	6,741 (19.7)	12,462 (19.5)	95,147 (22.0)	36,448 (12.1)	10,268 (14.7)	47,622 (21.4)	14,281 (17.3)	29,733 (15.6)	10,717 (15.7)	21,610 (20.8)	39,233 (18.1)
4	1763848 (18.6)	16,474 (21.1)	333,060 (24.9)	9,482 (24.2)	8,142 (24.5)	7,366 (21.5)	15,553 (24.4)	141,970 (32.9)	73,056 (24.2)	18,320 (26.2)	61,927 (27.9)	21,751 (26.3)	50,786 (26.7)	18,426 (27.0)	24,182 (23.3)	55,479 (25.6)
5 (most deprived)	1617403 (17.1)	13,623 (17.4)	326,070 (24.4)	13,280 (33.9)	10,913 (32.9)	7,710 (22.5)	16,268 (25.5)	87,151 (20.2)	161,060 (53.4)	33,197 (47.5)	56,803 (25.6)	33,005 (40.0)	78,211 (41.1)	26,640 (39.1)	16,023 (15.4)	66,414 (30.6)
**Number of people in household**																
1-2	4640170 (49.0)	40,771 (52.2)	488,759 (36.5)	15,990 (40.8)	10,726 (32.3)	11,902 (34.7)	23,799 (37.3)	97,231 (22.5)	42,428 (14.1)	9,345 (13.4)	60,595 (27.3)	38,377 (46.5)	56,075 (29.5)	23,712 (34.8)	45,953 (44.2)	72,641 (33.5)
3-5	3218856 (34.0)	23,852 (30.5)	519,471 (38.8)	16,297 (41.6)	13,849 (41.7)	14,185 (41.4)	25,233 (39.5)	195,776 (45.3)	113,234 (37.6)	29,054 (41.5)	92,868 (41.8)	30,397 (36.8)	78,979 (41.5)	26,167 (38.4)	32,700 (31.5)	84,887 (39.1)
6-10	291,873 (3.1)	3,784 (4.8)	148,123 (11.1)	2,813 (7.2)	4,623 (13.9)	3,560 (10.4)	7,187 (11.3)	73,063 (16.9)	89,761 (29.8)	20,303 (29.0)	38,185 (17.2)	5,477 (6.6)	34,666 (18.2)	10,535 (15.5)	8,602 (8.3)	31,239 (14.4)
11+	36,896 (0.4)	1,066 (1.4)	37,094 (2.8)	356 (0.9)	618 (1.9)	980 (2.9)	1,521 (2.4)	19,068 (4.4)	11,539 (3.8)	2,109 (3.0)	8,323 (3.7)	567 (0.7)	4,570 (2.4)	1,349 (2.0)	7,498 (7.2)	6,730 (3.1)
unknown	1273614 (13.5)	8,697 (11.1)	144,952 (10.8)	3,752 (9.6)	3,378 (10.2)	3,637 (10.6)	6,078 (9.5)	46,676 (10.8)	44,534 (14.8)	9,148 (13.1)	22,079 (9.9)	7,743 (9.4)	15,874 (8.3)	6,424 (9.4)	9,139 (8.8)	21,399 (9.9)
**Number of GP consultations in the 12 months prior to baseline**																
Mean (SD)	6.7 (9.1)	6.4 (9.6)	4.0 (7.1)	6.2 (9.1)	5.0 (7.9)	5.0 (8.2)	4.7 (8.3)	5.8 (8.3)	6.9 (9.5)	6.5 (8.5)	5.1 (7.9)	6.5 (8.6)	4.9 (7.7)	5.5 (8.7)	2.2 (4.9)	4.6 (7.9)
**Smoking status (missing set to non)**																
Never	4225276 (44.7)	32,469 (41.5)	625,154 (46.7)	18,438 (47.0)	22,133 (66.7)	20,818 (60.8)	34,974 (54.8)	336,178 (77.9)	210,718 (69.9)	46,360 (66.3)	157,778 (71.1)	43,734 (53.0)	145,279 (76.4)	43,971 (64.5)	82,208 (79.1)	126,242 (58.2)
Former	3626312 (38.3)	31,244 (40.0)	379,917 (28.4)	10,452 (26.7)	6,370 (19.2)	7,699 (22.5)	15,277 (23.9)	61,875 (14.3)	47,230 (15.7)	12,307 (17.6)	37,691 (17.0)	22,272 (27.0)	27,751 (14.6)	13,267 (19.5)	12,435 (12.0)	46,333 (21.4)
Current	1609821 (17.0)	14,457 (18.5)	333,328 (24.9)	10,318 (26.3)	4,691 (14.1)	5,747 (16.8)	13,567 (21.3)	33,761 (7.8)	43,548 (14.4)	11,292 (16.1)	26,581 (12.0)	16,555 (20.1)	17,134 (9.0)	10,949 (16.1)	9,249 (8.9)	44,321 (20.4)
**Body Mass Index (BMI, kg/m2)**																
Mean (SD)	27.6 (5.7)	26.9 (5.3)	26.2 (5.4)	27.6 (6.0)	27.7 (5.7)	25.7 (5.3)	26.3 (5.5)	26.3 (4.9)	27.5 (5.6)	26.2 (4.7)	25.9 (4.9)	28.4 (5.9)	27.9 (5.6)	27.9 (5.8)	22.9 (3.9)	26.3 (5.2)
**BMI with SA categories**																
Underweight	149,373 (1.6)	1,457 (1.9)	29,216 (2.2)	716 (1.8)	502 (1.5)	1,058 (3.1)	1,524 (2.4)	11,013 (2.6)	7,392 (2.5)	1,638 (2.3)	6,081 (2.7)	971 (1.2)	2,953 (1.6)	1,088 (1.6)	7,313 (7.0)	4,531 (2.1)
Normal	2739955 (29.0)	25,222 (32.3)	468,899 (35.0)	11,191 (28.5)	8,393 (25.3)	12,751 (37.2)	21,333 (33.4)	77,106 (17.9)	42,148 (14.0)	11,726 (16.8)	45,190 (20.4)	19,632 (23.8)	44,834 (23.6)	16,770 (24.6)	51,305 (49.4)	68,911 (31.8)
Overweight	2857872 (30.2)	24,039 (30.8)	336,958 (25.2)	10,301 (26.3)	8,952 (27.0)	8,310 (24.3)	15,338 (24.0)	139,189 (32.2)	82,589 (27.4)	23,219 (33.2)	68,481 (30.8)	25,188 (30.5)	52,978 (27.9)	19,104 (28.0)	15,321 (14.7)	56,142 (25.9)
Obese I	1474187 (15.6)	11,174 (14.3)	147,669 (11.0)	5,658 (14.4)	4,876 (14.7)	3,287 (9.6)	6,841 (10.7)	87,089 (20.2)	70,216 (23.3)	13,657 (19.5)	41,846 (18.8)	14,471 (17.5)	29,867 (15.7)	10,571 (15.5)	2,966 (2.9)	24,262 (11.2)
Obese II	575,733 (6.1)	3,710 (4.7)	51,830 (3.9)	2,381 (6.1)	1,895 (5.7)	1,087 (3.2)	2,462 (3.9)	26,631 (6.2)	28,633 (9.5)	3,853 (5.5)	12,311 (5.5)	5,978 (7.2)	11,272 (5.9)	4,133 (6.1)	630 (0.6)	7,725 (3.6)
Obese III	292,410 (3.1)	1,667 (2.1)	24,122 (1.8)	1,379 (3.5)	908 (2.7)	522 (1.5)	1,267 (2.0)	10,218 (2.4)	12,849 (4.3)	1,331 (1.9)	4,414 (2.0)	3,253 (3.9)	4,959 (2.6)	2,070 (3.0)	272 (0.3)	3,233 (1.5)
Unknown	1371879 (14.5)	10,901 (13.9)	279,705 (20.9)	7,582 (19.3)	7,668 (23.1)	7,249 (21.2)	15,053 (23.6)	80,568 (18.7)	57,669 (19.1)	14,535 (20.8)	43,727 (19.7)	13,068 (15.8)	43,301 (22.8)	14,451 (21.2)	26,085 (25.1)	52,092 (24.0)
**Glycated Haemoglobin (HbA1c)**																
HbA1c %, Mean (SD)	5.9 (1.0)	5.8 (0.9)	5.7 (0.9)	5.9 (1.1)	5.9 (1.1)	5.9 (1.0)	5.8 (1.0)	6.1 (1.1)	6.2 (1.3)	6.3 (1.3)	6.0 (1.1)	6.2 (1.3)	6.0 (1.2)	5.9 (1.2)	5.9 (0.8)	5.8 (1.0)
HbA1c mmo/mol, Mear	40.5 (60.8)	39.9 (9.8)	39.4 (78.0)	41.0 (12.5)	40.9 (13.1)	40.4 (11.4)	40.0 (11.3)	43.1 (12.4)	45.0 (235.8)	54.3 (660.8)	42.7 (106.3)	43.8 (13.9)	42.1 (136.8)	42.8 (222.4)	40.5 (9.3)	40.2 (11.1)
**HbA1c category**																
<6.5%	4112222 (43.5)	34,093 (43.6)	400,347 (29.9)	13,462 (34.3)	10,780 (32.5)	10,946 (31.9)	19,540 (30.6)	186,388 (43.2)	128,630 (42.7)	25,470 (36.4)	83,733 (37.7)	35,708 (43.3)	65,250 (34.3)	26,176 (38.4)	19,264 (18.5)	73,288 (33.8)
6.5-7.4%	323,922 (3.4)	2,473 (3.2)	23,842 (1.8)	1,262 (3.2)	948 (2.9)	888 (2.6)	1,402 (2.2)	28,893 (6.7)	19,513 (6.5)	5,049 (7.2)	10,930 (4.9)	6,031 (7.3)	6,775 (3.6)	2,488 (3.6)	1,928 (1.9)	5,752 (2.7)
7.5-7.9%	87,173 (0.9)	656 (0.8)	6,329 (0.5)	316 (0.8)	217 (0.7)	230 (0.7)	381 (0.6)	7,323 (1.7)	5,597 (1.9)	1,426 (2.0)	2,771 (1.2)	1,318 (1.6)	1,493 (0.8)	565 (0.8)	412 (0.4)	1,413 (0.7)
8-8.9%	95,448 (1.0)	642 (0.8)	6,972 (0.5)	332 (0.8)	240 (0.7)	270 (0.8)	384 (0.6)	7,379 (1.7)	6,478 (2.1)	1,582 (2.3)	2,873 (1.3)	1,349 (1.6)	1,643 (0.9)	566 (0.8)	357 (0.3)	1,608 (0.7)
>=9%	106,939 (1.1)	623 (0.8)	8,348 (0.6)	464 (1.2)	352 (1.1)	332 (1.0)	538 (0.8)	7,778 (1.8)	8,331 (2.8)	1,660 (2.4)	3,291 (1.5)	1,894 (2.3)	2,528 (1.3)	993 (1.5)	289 (0.3)	1,926 (0.9)
Unknown	4735705 (50.1)	39,683 (50.8)	892,561 (66.7)	23,372 (59.6)	20,657 (62.2)	21,598 (63.0)	41,573 (65.1)	194,053 (44.9)	132,947 (44.1)	34,772 (49.7)	118,452 (53.3)	36,261 (43.9)	112,475 (59.1)	37,399 (54.8)	81,642 (78.6)	132,909 (61.3)
**Blood Pressure**																
Systolic BP, Mean (SD)	128.5 (15.4)	128.7 (15.9)	125.6 (15.6)	125.4 (15.4)	125.9 (15.5)	122.0 (15.0)	123.0 (15.2)	125.1 (15.5)	122.9 (15.2)	121.5 (15.0)	123.1 (15.1)	129.7 (16.1)	126.1 (15.9)	126.0 (16.0)	120.4 (15.6)	123.1 (15.3)
Diastolic BP, Mean (SD)	76.8 (9.8)	76.3 (9.9)	76.7 (10.1)	76.9 (10.2)	77.5 (10.2)	75.7 (9.9)	76.2 (10.1)	76.9 (9.7)	76.2 (9.6)	76.1 (9.8)	76.4 (9.8)	78.4 (10.3)	77.9 (10.3)	77.8 (10.4)	74.8 (10.0)	76.1 (9.9)
**Blood pressure four levels non-missing**																
Normal	1497296 (15.8)	12,377 (15.8)	209,735 (15.7)	7,562 (19.3)	5,505 (16.6)	7,296 (21.3)	12,348 (19.3)	79,664 (18.4)	68,793 (22.8)	17,455 (25.0)	45,183 (20.3)	12,224 (14.8)	32,219 (16.9)	12,343 (18.1)	15,902 (15.3)	41,573 (19.2)
Elevated	1103213 (11.7)	8,540 (10.9)	114,007 (8.5)	4,094 (10.4)	2,897 (8.7)	3,103 (9.1)	5,619 (8.8)	40,439 (9.4)	31,675 (10.5)	6,883 (9.8)	20,294 (9.1)	8,643 (10.5)	17,108 (9.0)	6,342 (9.3)	5,315 (5.1)	18,283 (8.4)
High Stage I	2600655 (27.5)	19,636 (25.1)	259,844 (19.4)	9,003 (23.0)	7,232 (21.8)	6,682 (19.5)	12,237 (19.2)	102,809 (23.8)	71,601 (23.7)	16,385 (23.4)	48,167 (21.7)	22,684 (27.5)	41,924 (22.0)	15,997 (23.5)	11,840 (11.4)	41,618 (19.2)
High Stage II	1850569 (19.6)	14,717 (18.8)	160,109 (12.0)	5,444 (13.9)	4,389 (13.2)	3,038 (8.9)	6,137 (9.6)	56,534 (13.1)	33,732 (11.2)	7,178 (10.3)	22,957 (10.3)	17,554 (21.3)	26,445 (13.9)	10,018 (14.7)	5,436 (5.2)	20,997 (9.7)
Unknown	2409676 (25.5)	22,900 (29.3)	594,704 (44.4)	13,105 (33.4)	13,171 (39.7)	14,145 (41.3)	27,477 (43.1)	152,368 (35.3)	95,695 (31.7)	22,058 (31.5)	85,449 (38.5)	21,456 (26.0)	72,468 (38.1)	23,487 (34.4)	65,399 (62.9)	94,425 (43.5)
**Co-morbidities**																
T1DM	54,986 (0.6)	344 (0.4)	4,610 (0.3)	198 (0.5)	120 (0.4)	109 (0.3)	210 (0.3)	1,097 (0.3)	868 (0.3)	123 (0.2)	420 (0.2)	420 (0.5)	687 (0.4)	256 (0.4)	79 (0.1)	558 (0.3)
T2DM	691,952 (7.3)	5,165 (6.6)	50,681 (3.8)	2,665 (6.8)	1,975 (5.9)	1,943 (5.7)	3,020 (4.7)	58,726 (13.6)	43,616 (14.5)	10,975 (15.7)	22,424 (10.1)	12,441 (15.1)	14,061 (7.4)	5,088 (7.5)	3,387 (3.3)	12,140 (5.6)
Diagnosed hypertensio	2251054 (23.8)	19,492 (24.9)	167,025 (12.5)	6,208 (15.8)	4,834 (14.6)	3,330 (9.7)	6,340 (9.9)	84,589 (19.6)	45,575 (15.1)	10,229 (14.6)	32,844 (14.8)	26,201 (31.7)	31,665 (16.7)	11,658 (17.1)	7,213 (6.9)	24,493 (11.3)
Chronic Cardiac Disease	748,328 (7.9)	6,848 (8.8)	48,396 (3.6)	1,320 (3.4)	727 (2.2)	993 (2.9)	1,691 (2.6)	24,785 (5.7)	17,594 (5.8)	3,331 (4.8)	8,193 (3.7)	5,173 (6.3)	4,047 (2.1)	1,872 (2.7)	1,419 (1.4)	6,717 (3.1)
Stroke	233,301 (2.5)	2,304 (2.9)	14,137 (1.1)	491 (1.3)	259 (0.8)	274 (0.8)	471 (0.7)	5,760 (1.3)	3,902 (1.3)	797 (1.1)	1,970 (0.9)	2,122 (2.6)	1,527 (0.8)	724 (1.1)	470 (0.5)	1,691 (0.8)
CKD defined by egfr<60	609,546 (6.4)	5,293 (6.8)	32,246 (2.4)	1,597 (4.1)	785 (2.4)	492 (1.4)	913 (1.4)	13,499 (3.1)	8,818 (2.9)	1,633 (2.3)	4,029 (1.8)	8,315 (10.1)	4,439 (2.3)	2,288 (3.4)	952 (0.9)	3,830 (1.8)
end stage renal failure	13,494 (0.1)	109 (0.1)	1,012 (0.1)	73 (0.2)	38 (0.1)	37 (0.1)	71 (0.1)	1,082 (0.3)	798 (0.3)	137 (0.2)	393 (0.2)	329 (0.4)	389 (0.2)	160 (0.2)	81 (0.1)	271 (0.1)
Cancer	637,162 (6.7)	5,682 (7.3)	41,980 (3.1)	1,075 (2.7)	674 (2.0)	665 (1.9)	1,304 (2.0)	9,429 (2.2)	5,383 (1.8)	906 (1.3)	4,150 (1.9)	4,452 (5.4)	3,188 (1.7)	1,609 (2.4)	1,760 (1.7)	4,880 (2.2)
Autoimmune disease	567,942 (6.0)	5,067 (6.5)	42,123 (3.1)	1,260 (3.2)	649 (2.0)	1,172 (3.4)	1,907 (3.0)	16,621 (3.8)	11,607 (3.8)	2,689 (3.8)	6,431 (2.9)	2,681 (3.2)	2,615 (1.4)	1,616 (2.4)	1,763 (1.7)	5,741 (2.6)
Immunosuppressed	47,659 (0.5)	545 (0.7)	5,939 (0.4)	404 (1.0)	1,051 (3.2)	163 (0.5)	500 (0.8)	1,976 (0.5)	1,354 (0.4)	207 (0.3)	1,065 (0.5)	1,329 (1.6)	7,296 (3.8)	1,462 (2.1)	236 (0.2)	1,189 (0.5)
Chronic liver disease	62,097 (0.7)	765 (1.0)	7,039 (0.5)	152 (0.4)	280 (0.8)	184 (0.5)	278 (0.4)	2,055 (0.5)	2,051 (0.7)	341 (0.5)	1,230 (0.6)	396 (0.5)	2,094 (1.1)	545 (0.8)	1,174 (1.1)	1,271 (0.6)
Dementia	21,303 (0.2)	319 (0.4)	1,233 (0.1)	60 (0.2)	12 (0.0)	10 (0.0)	24 (0.0)	554 (0.1)	371 (0.1)	110 (0.2)	164 (0.1)	360 (0.4)	119 (0.1)	59 (0.1)	40 (0.0)	155 (0.1)
Neurological disease	108,466 (1.1)	836 (1.1)	6,998 (0.5)	277 (0.7)	121 (0.4)	170 (0.5)	320 (0.5)	2,504 (0.6)	2,119 (0.7)	274 (0.4)	906 (0.4)	808 (1.0)	728 (0.4)	415 (0.6)	204 (0.2)	959 (0.4)
Asthma	1677850 (17.7)	10,816 (13.8)	102,309 (7.6)	8,502 (21.7)	3,535 (10.6)	5,101 (14.9)	8,638 (13.5)	49,090 (11.4)	45,007 (14.9)	9,301 (13.3)	22,347 (10.1)	14,241 (17.2)	14,405 (7.6)	8,705 (12.8)	4,927 (4.7)	17,479 (8.1)
Chronic Respiratory Dis	494,413 (5.2)	5,374 (6.9)	27,478 (2.1)	665 (1.7)	328 (1.0)	460 (1.3)	821 (1.3)	6,999 (1.6)	6,546 (2.2)	1,078 (1.5)	2,812 (1.3)	2,355 (2.9)	1,662 (0.9)	986 (1.4)	561 (0.5)	2,959 (1.4)

Table S2. Comparison of characteristics of the general population vs. those receiving a test for SARS-CoV-2 in waves 1 and 2

		Wave 1		Wave 2	
		Denominator	Tested Population	Denominator	Tested Population
		17,288,532	1,700,196	17,636,366	2,647,756
Age (years)	Mean (SD)	49.8 (18.7)	51.3 (19.9)	49.7 (18.7)	46.6 (18.5)
Sex	Male	49.9%	41.4%	50	44.0%
Ethnic group	White	64.3%	66.8%	64.2%	65.5%
	South Asian	5.9%	6.2%	6.0%	6.3%
	Black	2.0%	2.0%	2.0%	1.8%
	Mixed	1.0%	1.0%	1.0%	1.0%
	Other	1.9%	1.2%	1.9%	1.2%
	Unknown	25.0%	22.7%	24.8%	24.2%
Index of Multiple Deprivation Quintile	Q1	20.4%	19.4%	20.4%	20.7%
	Q2	20.1%	19.7%	20.1%	19.8%
	Q3	20.1%	20.2%	20.4%	19.9%
	Q4	20.0%	20.8%	20.0%	20.0%
	Q5	19.3%	19.9%	19.1%	19.5%
Geographic Region	East Midlands	23.2%	23.1%	23.2%	24.7%
	East of England	17.5%	19.2%	17.5%	17.1%
	London	6.8%	5.3%	7.0%	6.1%
	North East	4.6%	5.6%	4.6%	4.9%
	North West	8.9%	8.5%	8.8%	9.9%
	South East	6.7%	6.9%	6.7%	5.9%
	West Midlands	13.9%	14.1%	14.0%	11.7%
	Yorkshire/Humber	4.0%	3.6%	4.0%	4.4%
Count of co-morbid conditions	Mean (SD)	0.6 (0.9)	0.9 (1.1)	0.6 (0.9)	0.7 (0.9)

Table S3. Association between ethnicity in 5 categories and non-COVID-19 related mortality wave 1 (serial adjustment)

	Denominator	Event	Total person-weeks	Rate per 1,000 person-weeks	Crude		Age-Sex Adjusted		plus Deprivation		Plus Co-morbidities and Clinical Factors		plus household size	
					HR	95% CI	HR	95% CI	HR	95% CI	HR	95% CI	HR	95% CI
Non COVID-19 mortality (n=71,920)														
White	10,877,978	48,100	285,149,111	0.17	1		1		1		1		1	
South Asian	1,025,319	1,723	26,916,194	0.06	0.42	(0.40 - 0.44)	0.87	(0.83 - 0.91)	0.79	(0.75 - 0.83)	0.91	(0.86 - 0.95)	0.85	(0.81 - 0.90)
Black	340,912	606	8,948,563	0.07	0.45	(0.41 - 0.48)	0.89	(0.82 - 0.97)	0.78	(0.72 - 0.85)	0.85	(0.78 - 0.92)	0.85	(0.78 - 0.92)
Mixed	170,484	178	4,477,775	0.04	0.26	(0.22 - 0.30)	0.8	(0.69 - 0.92)	0.74	(0.64 - 0.86)	0.81	(0.70 - 0.94)	0.81	(0.70 - 0.93)
Other	320,788	296	8,426,262	0.04	0.23	(0.21 - 0.26)	0.66	(0.59 - 0.74)	0.63	(0.56 - 0.70)	0.69	(0.62 - 0.77)	0.68	(0.61 - 0.77)
Unknown	4,553,051	21,017	119,337,957	0.18	1.04	(1.02 - 1.06)	0.98	(0.97 - 1.00)	0.99	(0.97 - 1.01)	1.05	(1.04 - 1.07)	1.06	(1.04 - 1.08)

*Any counts <5 are set to 5 per data disclosure agreements

Table S4. Association between ethnicity in 16 categories and COVID-19 outcomes in wave 1 (serial adjustment)

	Denominator	Event	Crude		Age-Sex Adjusted		plus Deprivation		Plus Co-morbidities and Clinical Factors		plus household size	
			HR	95% CI	HR	95% CI	HR	95% CI	HR	95% CI	HR	95% CI
tested		1216801										
British	9461409	713121	1		1		1		1		1	
Irish	78170	6160	1.08	(1.06 - 1.11)	1.06	(1.04 - 1.09)	1.05	(1.03 - 1.08)	1.06	(1.04 - 1.09)	1.06	(1.04 - 1.09)
Other White	1338399	73900	0.74	(0.73 - 0.75)	0.74	(0.74 - 0.75)	0.73	(0.73 - 0.74)	0.81	(0.80 - 0.81)	0.8	(0.80 - 0.81)
White + Caribbean	39208	2838	0.97	(0.94 - 1.01)	0.99	(0.95 - 1.03)	0.96	(0.93 - 1.00)	0.96	(0.92 - 0.99)	0.96	(0.92 - 0.99)
White + African	33194	2619	1.06	(1.02 - 1.10)	1.1	(1.06 - 1.14)	1.07	(1.03 - 1.11)	1.15	(1.11 - 1.19)	1.15	(1.10 - 1.19)
White + Asian	34264	2608	1.01	(0.97 - 1.05)	1.04	(1.00 - 1.08)	1.03	(1.00 - 1.08)	1.08	(1.04 - 1.12)	1.07	(1.03 - 1.12)
Other mixed	63818	4061	0.86	(0.84 - 0.89)	0.88	(0.86 - 0.91)	0.87	(0.84 - 0.90)	0.92	(0.89 - 0.95)	0.91	(0.89 - 0.94)
Indian	431814	40231	1.13	(1.12 - 1.14)	1.15	(1.14 - 1.17)	1.13	(1.12 - 1.14)	1.17	(1.16 - 1.18)	1.15	(1.14 - 1.16)
Pakistani	301496	21952	0.99	(0.98 - 1.01)	1.02	(1.00 - 1.03)	0.97	(0.96 - 0.99)	0.95	(0.94 - 0.96)	0.94	(0.93 - 0.96)
Bangladeshi	69959	3924	0.73	(0.70 - 0.75)	0.75	(0.72 - 0.77)	0.71	(0.69 - 0.74)	0.7	(0.68 - 0.72)	0.7	(0.67 - 0.72)
Other Asian	222050	16540	1.01	(1.00 - 1.03)	1.04	(1.02 - 1.05)	1.01	(1.00 - 1.03)	1.07	(1.05 - 1.08)	1.06	(1.04 - 1.07)
Caribbean	82561	5972	1	(0.97 - 1.03)	1	(0.98 - 1.03)	0.97	(0.94 - 0.99)	0.97	(0.95 - 1.00)	0.97	(0.95 - 1.00)
African	190164	14623	1.02	(1.00 - 1.04)	1.06	(1.04 - 1.08)	1.02	(1.00 - 1.04)	1.1	(1.08 - 1.12)	1.09	(1.08 - 1.11)
Other Black	68187	4710	0.93	(0.90 - 0.96)	0.97	(0.94 - 1.00)	0.93	(0.91 - 0.96)	0.97	(0.94 - 0.99)	0.96	(0.94 - 0.99)
Chinese	103892	2899	0.36	(0.34 - 0.37)	0.37	(0.35 - 0.38)	0.37	(0.35 - 0.38)	0.46	(0.44 - 0.47)	0.45	(0.43 - 0.46)

Other	216896	12925	0.8	(0.79 - 0.82)	0.83	(0.81 - 0.84)	0.81	(0.80 - 0.82)	0.88	(0.86 - 0.89)	0.87	(0.86 - 0.89)
Unknown	4553051	287718	0.84	(0.83 - 0.84)	0.86	(0.85 - 0.86)	0.86	(0.85 - 0.86)	0.95	(0.95 - 0.96)	0.95	(0.95 - 0.96)
positivetest		71246										
British	9461409	36214	1		1		1		1		1	
Irish	78170	335	1.24	(1.11 - 1.38)	1.17	(1.05 - 1.30)	1.14	(1.03 - 1.27)	1.16	(1.04 - 1.29)	1.15	(1.04 - 1.29)
Other White	1338399	4631	0.99	(0.96 - 1.02)	1.09	(1.06 - 1.13)	1.04	(1.01 - 1.08)	1.15	(1.12 - 1.19)	1.12	(1.08 - 1.16)
White + Caribbean	39208	171	1.17	(1.01 - 1.36)	1.35	(1.16 - 1.57)	1.26	(1.09 - 1.47)	1.25	(1.07 - 1.45)	1.24	(1.07 - 1.45)
White + African	33194	223	1.82	(1.60 - 2.08)	2.16	(1.89 - 2.46)	2.01	(1.76 - 2.29)	1.99	(1.74 - 2.27)	1.92	(1.69 - 2.20)
White + Asian	34264	192	1.55	(1.34 - 1.78)	1.84	(1.60 - 2.12)	1.8	(1.56 - 2.08)	1.82	(1.58 - 2.10)	1.77	(1.53 - 2.04)
Other mixed	63818	254	1.15	(1.01 - 1.30)	1.36	(1.20 - 1.54)	1.3	(1.15 - 1.47)	1.35	(1.19 - 1.53)	1.32	(1.17 - 1.49)
Indian	431814	4035	2.36	(2.28 - 2.45)	2.6	(2.51 - 2.69)	2.46	(2.37 - 2.55)	2.24	(2.17 - 2.33)	2.08	(2.00 - 2.15)
Pakistani	301496	3466	2.65	(2.56 - 2.75)	3.02	(2.91 - 3.13)	2.7	(2.60 - 2.80)	2.29	(2.21 - 2.38)	2.07	(1.99 - 2.15)
Bangladeshi	69959	366	1.33	(1.20 - 1.48)	1.55	(1.39 - 1.72)	1.38	(1.24 - 1.53)	1.2	(1.08 - 1.33)	1.1	(0.99 - 1.22)
Other Asian	222050	1812	2.32	(2.21 - 2.43)	2.66	(2.54 - 2.79)	2.51	(2.40 - 2.64)	2.38	(2.27 - 2.50)	2.24	(2.13 - 2.35)
Caribbean	82561	530	1.78	(1.64 - 1.94)	1.8	(1.65 - 1.96)	1.64	(1.50 - 1.78)	1.54	(1.41 - 1.68)	1.53	(1.40 - 1.67)
African	190164	1390	1.95	(1.85 - 2.06)	2.31	(2.19 - 2.44)	2.09	(1.97 - 2.20)	2	(1.89 - 2.11)	1.91	(1.80 - 2.02)
Other Black	68187	366	1.48	(1.33 - 1.64)	1.74	(1.57 - 1.93)	1.59	(1.43 - 1.76)	1.53	(1.38 - 1.69)	1.48	(1.33 - 1.64)
Chinese	103892	140	0.37	(0.31 - 0.44)	0.45	(0.38 - 0.53)	0.45	(0.38 - 0.53)	0.52	(0.44 - 0.61)	0.49	(0.42 - 0.58)
Other	216896	1073	1.4	(1.31 - 1.48)	1.62	(1.52 - 1.72)	1.52	(1.43 - 1.62)	1.6	(1.50 - 1.70)	1.53	(1.44 - 1.63)
Unknown	4553051	16048	0.97	(0.95 - 0.99)	1	(0.99 - 1.02)	1.01	(0.99 - 1.02)	1.08	(1.06 - 1.10)	1.07	(1.05 - 1.09)
Hospital Admission (n=32,473)												
British	9683276	18627	1		1		1		1		1	
Irish	79945	257	1.47	(1.30 - 1.67)	1.23	(1.09 - 1.40)	1.16	(1.03 - 1.31)	1.14	(1.01 - 1.29)	1.14	(1.00 - 1.29)
Other White	1347091	1620	0.58	(0.55 - 0.61)	1.01	(0.96 - 1.06)	0.96	(0.91 - 1.01)	1.06	(1.00 - 1.11)	1.04	(0.99 - 1.10)
White + Caribbean	39631	79	0.87	(0.70 - 1.08)	1.68	(1.35 - 2.09)	1.49	(1.20 - 1.87)	1.43	(1.15 - 1.79)	1.43	(1.14 - 1.78)
White + African	33438	65	0.9	(0.70 - 1.14)	2.03	(1.59 - 2.59)	1.81	(1.42 - 2.32)	1.92	(1.50 - 2.45)	1.86	(1.46 - 2.38)
White + Asian	34629	63	0.87	(0.68 - 1.11)	2.03	(1.59 - 2.61)	1.96	(1.53 - 2.51)	2.02	(1.58 - 2.59)	1.98	(1.55 - 2.54)
Other mixed	64235	95	0.68	(0.56 - 0.84)	1.61	(1.32 - 1.97)	1.51	(1.23 - 1.85)	1.58	(1.29 - 1.94)	1.55	(1.27 - 1.90)
Indian	432760	1179	1.14	(1.08 - 1.22)	1.75	(1.65 - 1.86)	1.62	(1.52 - 1.73)	1.48	(1.39 - 1.58)	1.41	(1.32 - 1.50)
Pakistani	300528	924	1.29	(1.21 - 1.38)	2.47	(2.31 - 2.64)	2.07	(1.93 - 2.22)	1.65	(1.54 - 1.76)	1.48	(1.38 - 1.59)
Bangladeshi	70395	153	0.89	(0.76 - 1.04)	1.97	(1.68 - 2.31)	1.63	(1.39 - 1.92)	1.34	(1.14 - 1.57)	1.21	(1.03 - 1.42)
Other Asian	222868	580	1.17	(1.07 - 1.27)	2.23	(2.05 - 2.43)	2.05	(1.88 - 2.23)	1.95	(1.79 - 2.12)	1.85	(1.70 - 2.02)

Caribbean	83042	393	1.92	(1.74 - 2.13)	1.96	(1.77 - 2.17)	1.68	(1.52 - 1.86)	1.58	(1.43 - 1.75)	1.58	(1.43 - 1.75)
African	190395	499	1.12	(1.02 - 1.23)	2.51	(2.29 - 2.75)	2.15	(1.96 - 2.35)	2.19	(2.00 - 2.40)	2.1	(1.91 - 2.30)
Other Black	69124	159	0.98	(0.84 - 1.15)	1.98	(1.69 - 2.31)	1.72	(1.47 - 2.01)	1.67	(1.43 - 1.96)	1.63	(1.39 - 1.90)
Chinese	104560	70	0.31	(0.25 - 0.39)	0.76	(0.60 - 0.96)	0.75	(0.60 - 0.95)	1	(0.79 - 1.26)	0.97	(0.77 - 1.23)
Other	218969	434	0.91	(0.83 - 1.01)	1.82	(1.65 - 2.00)	1.67	(1.52 - 1.85)	1.79	(1.62 - 1.97)	1.74	(1.58 - 1.91)
Unknown	4408386	7276	0.86	(0.84 - 0.89)	0.97	(0.94 - 0.99)	0.97	(0.94 - 0.99)	1.07	(1.04 - 1.10)	1.07	(1.04 - 1.10)
ICU admission (N=3,091)												
British	9461409	1500	1		1		1		1		1	
Irish	78170	10	0.37	(0.15 - 0.88)	0.37	(0.15 - 0.88)	0.36	(0.15 - 0.86)	0.38	(0.16 - 0.91)	0.38	(0.16 - 0.91)
Other White	1338399	190	0.83	(0.71 - 0.97)	1.31	(1.12 - 1.53)	1.25	(1.07 - 1.47)	1.46	(1.25 - 1.71)	1.43	(1.23 - 1.68)
White + Caribbean	39208	11	1.61	(0.89 - 2.91)	2.63	(1.45 - 4.77)	2.44	(1.34 - 4.42)	2.34	(1.29 - 4.24)	2.32	(1.28 - 4.21)
White + African	33194	21	3.6	(2.34 - 5.55)	5.72	(3.71 - 8.81)	5.25	(3.40 - 8.09)	5.08	(3.30 - 7.84)	4.93	(3.19 - 7.60)
White + Asian	34264	10	1.74	(0.94 - 3.25)	3.18	(1.70 - 5.92)	3.1	(1.66 - 5.77)	3.39	(1.82 - 6.32)	3.31	(1.77 - 6.17)
Other mixed	63818	14	1.27	(0.75 - 2.15)	2.34	(1.38 - 3.97)	2.23	(1.31 - 3.78)	2.39	(1.41 - 4.06)	2.34	(1.38 - 3.97)
Indian	431814	161	2.19	(1.84 - 2.60)	2.93	(2.46 - 3.48)	2.78	(2.33 - 3.30)	2.23	(1.87 - 2.67)	2.1	(1.76 - 2.52)
Pakistani	301496	118	2.21	(1.82 - 2.68)	3.54	(2.92 - 4.30)	3.13	(2.57 - 3.81)	2.21	(1.81 - 2.69)	2.03	(1.65 - 2.49)
Bangladeshi	69959	25	1.96	(1.31 - 2.91)	3.51	(2.35 - 5.22)	3.08	(2.06 - 4.60)	2.46	(1.65 - 3.68)	2.27	(1.52 - 3.41)
Other Asian	222050	106	2.71	(2.21 - 3.31)	4.18	(3.41 - 5.14)	3.93	(3.20 - 4.83)	3.36	(2.73 - 4.14)	3.19	(2.59 - 3.94)
Caribbean	82561	41	2.66	(1.94 - 3.65)	2.77	(2.02 - 3.79)	2.5	(1.83 - 3.43)	2.31	(1.68 - 3.17)	2.3	(1.68 - 3.16)
African	190164	118	3.39	(2.80 - 4.11)	5.26	(4.33 - 6.38)	4.69	(3.86 - 5.71)	4.37	(3.58 - 5.33)	4.2	(3.44 - 5.12)
Other Black	68187	27	2.15	(1.47 - 3.16)	3.05	(2.08 - 4.48)	2.77	(1.89 - 4.07)	2.62	(1.78 - 3.84)	2.55	(1.74 - 3.75)
Chinese	103892	18	1.01	(0.64 - 1.62)	2.03	(1.27 - 3.24)	2.02	(1.27 - 3.23)	3.3	(2.06 - 5.26)	3.21	(2.01 - 5.12)
Other	216896	79	2.12	(1.68 - 2.67)	3.4	(2.70 - 4.29)	3.2	(2.53 - 4.04)	3.52	(2.79 - 4.45)	3.41	(2.70 - 4.31)
Unknown	4553051	662	0.92	(0.84 - 1.01)	1.05	(0.96 - 1.15)	1.05	(0.96 - 1.15)	1.12	(1.02 - 1.23)	1.12	(1.02 - 1.23)
onscoviddeath		11649										
British	9461409	6992	1		1		1		1		1	
Irish	78170	86	1.31	(1.06 - 1.62)	1.03	(0.83 - 1.27)	0.96	(0.77 - 1.19)	0.92	(0.74 - 1.14)	0.92	(0.74 - 1.14)
Other White	1338399	436	0.41	(0.37 - 0.45)	0.9	(0.82 - 1.00)	0.87	(0.79 - 0.96)	0.91	(0.83 - 1.01)	0.91	(0.82 - 1.00)
White + Caribbean	39208	28	0.83	(0.57 - 1.20)	2.02	(1.39 - 2.93)	1.77	(1.22 - 2.57)	1.62	(1.11 - 2.35)	1.62	(1.12 - 2.36)
White + African	33194	9	0.33	(0.17 - 0.63)	1.32	(0.69 - 2.54)	1.18	(0.61 - 2.27)	1.31	(0.68 - 2.52)	1.26	(0.65 - 2.42)
White + Asian	34264	9	0.33	(0.17 - 0.63)	1.25	(0.65 - 2.41)	1.2	(0.62 - 2.31)	1.23	(0.64 - 2.36)	1.21	(0.63 - 2.33)
Other mixed	63818	19	0.35	(0.23 - 0.55)	1.37	(0.87 - 2.16)	1.28	(0.81 - 2.01)	1.33	(0.84 - 2.08)	1.31	(0.84 - 2.06)

Indian	431814	335	0.86	(0.76 - 0.96)	1.59	(1.42 - 1.79)	1.46	(1.30 - 1.64)	1.41	(1.25 - 1.58)	1.29	(1.14 - 1.45)
Pakistani	301496	210	0.79	(0.69 - 0.91)	2.07	(1.80 - 2.38)	1.69	(1.47 - 1.94)	1.35	(1.17 - 1.55)	1.03	(0.89 - 1.19)
Bangladeshi	69959	49	0.78	(0.59 - 1.03)	2.7	(2.04 - 3.59)	2.19	(1.65 - 2.91)	1.68	(1.26 - 2.22)	1.29	(0.97 - 1.71)
Other Asian	222050	140	0.72	(0.60 - 0.85)	1.96	(1.65 - 2.33)	1.79	(1.51 - 2.13)	1.72	(1.45 - 2.05)	1.57	(1.32 - 1.87)
Caribbean	82561	137	1.74	(1.46 - 2.06)	1.65	(1.39 - 1.96)	1.4	(1.18 - 1.67)	1.27	(1.07 - 1.51)	1.33	(1.12 - 1.58)
African	190164	91	0.53	(0.43 - 0.66)	2.17	(1.76 - 2.68)	1.84	(1.49 - 2.27)	1.86	(1.51 - 2.30)	1.77	(1.43 - 2.19)
Other Black	68187	40	0.64	(0.47 - 0.88)	2.05	(1.50 - 2.80)	1.77	(1.29 - 2.42)	1.67	(1.22 - 2.28)	1.61	(1.18 - 2.20)
Chinese	103892	20	0.24	(0.15 - 0.37)	0.88	(0.57 - 1.37)	0.86	(0.55 - 1.33)	1.03	(0.67 - 1.61)	1.05	(0.67 - 1.63)
Other	216896	87	0.47	(0.38 - 0.58)	1.35	(1.09 - 1.68)	1.25	(1.01 - 1.55)	1.28	(1.03 - 1.59)	1.26	(1.01 - 1.56)
Unknown	4553051	2961	0.88	(0.84 - 0.92)	0.91	(0.87 - 0.95)	0.92	(0.88 - 0.96)	0.99	(0.95 - 1.04)	1.01	(0.96 - 1.05)
**ons_noncoviddeath**		**71920**										
British	9461409	45049	1		1		1		1		1	
Irish	78170	475	1.39	(1.27 - 1.53)	1.13	(1.03 - 1.24)	1.07	(0.98 - 1.18)	1	(0.92 - 1.10)	1	(0.91 - 1.09)
Other White	1338399	2576	0.43	(0.42 - 0.45)	0.92	(0.89 - 0.96)	0.9	(0.86 - 0.93)	0.94	(0.90 - 0.97)	0.93	(0.89 - 0.97)
White + Caribbean	39208	75	0.42	(0.34 - 0.53)	1.06	(0.84 - 1.33)	0.95	(0.76 - 1.19)	1	(0.80 - 1.25)	1	(0.80 - 1.26)
White + African	33194	30	0.2	(0.14 - 0.29)	0.78	(0.54 - 1.11)	0.71	(0.50 - 1.02)	0.86	(0.60 - 1.23)	0.84	(0.59 - 1.21)
White + Asian	34264	25	0.16	(0.11 - 0.24)	0.62	(0.42 - 0.91)	0.6	(0.40 - 0.89)	0.65	(0.44 - 0.96)	0.64	(0.43 - 0.95)
Other mixed	63818	48	0.17	(0.13 - 0.23)	0.64	(0.48 - 0.85)	0.61	(0.46 - 0.81)	0.66	(0.50 - 0.88)	0.66	(0.49 - 0.87)
Indian	431814	846	0.45	(0.42 - 0.48)	0.84	(0.78 - 0.90)	0.78	(0.73 - 0.84)	0.95	(0.88 - 1.02)	0.92	(0.85 - 0.98)
Pakistani	301496	523	0.38	(0.35 - 0.42)	0.96	(0.88 - 1.05)	0.81	(0.74 - 0.88)	0.87	(0.80 - 0.95)	0.78	(0.71 - 0.85)
Bangladeshi	69959	91	0.29	(0.24 - 0.36)	0.97	(0.79 - 1.19)	0.82	(0.66 - 1.00)	0.82	(0.67 - 1.01)	0.73	(0.59 - 0.90)
Other Asian	222050	263	0.28	(0.25 - 0.31)	0.77	(0.68 - 0.87)	0.71	(0.63 - 0.81)	0.86	(0.76 - 0.97)	0.83	(0.74 - 0.94)
Caribbean	82561	346	0.96	(0.86 - 1.06)	0.93	(0.83 - 1.03)	0.81	(0.73 - 0.90)	0.84	(0.75 - 0.93)	0.85	(0.76 - 0.95)
African	190164	161	0.19	(0.17 - 0.23)	0.76	(0.65 - 0.89)	0.67	(0.57 - 0.78)	0.82	(0.71 - 0.96)	0.8	(0.68 - 0.93)
Other Black	68187	99	0.33	(0.27 - 0.40)	0.99	(0.81 - 1.21)	0.88	(0.72 - 1.07)	0.91	(0.75 - 1.11)	0.89	(0.73 - 1.09)
Chinese	103892	56	0.12	(0.09 - 0.16)	0.44	(0.34 - 0.57)	0.43	(0.33 - 0.56)	0.52	(0.40 - 0.68)	0.52	(0.40 - 0.68)
Other	216896	240	0.25	(0.22 - 0.29)	0.73	(0.65 - 0.83)	0.69	(0.61 - 0.78)	0.74	(0.65 - 0.84)	0.73	(0.64 - 0.83)
Unknown	4553051	21017	0.98	(0.96 - 0.99)	0.98	(0.96 - 0.99)	0.99	(0.97 - 1.00)	1.05	(1.03 - 1.07)	1.06	(1.04 - 1.08)

*Any counts <5 are set to 5 per data disclosure agreements

Table S5. Association between ethnicity in 5 categories and COVID-19 outcomes in wave 2 (serial adjustment)

	Denominator	Event	Crude		Age-Sex Adjusted		Plus Deprivation		Plus Co-morbidities and Clinical Factors		Plus Household size	
			HR	95% CI	HR	95% CI	HR	95% CI	HR	95% CI	HR	95% CI
**Tested (n=2,647,756)**												
White	11323083	1733272	1		1		1		1		1	
South Asian	1061336	166856	1.03	(1.02 - 1.03)	0.96	(0.95 - 0.96)	0.97	(0.96 - 0.97)	0.96	(0.95 - 0.96)	0.96	(0.95 - 0.96)
Black	355625	48068	0.86	(0.85 - 0.87)	0.81	(0.80 - 0.81)	0.82	(0.81 - 0.83)	0.83	(0.82 - 0.84)	0.83	(0.82 - 0.84)
Mixed	179557	26679	0.98	(0.96 - 0.99)	0.87	(0.86 - 0.88)	0.88	(0.87 - 0.89)	0.89	(0.88 - 0.90)	0.9	(0.88 - 0.91)
Other	334962	32904	0.64	(0.64 - 0.65)	0.58	(0.58 - 0.59)	0.58	(0.58 - 0.59)	0.64	(0.63 - 0.64)	0.64	(0.64 - 0.65)
Unknown	4381803	639977	0.95	(0.94 - 0.95)	0.92	(0.92 - 0.92)	0.92	(0.92 - 0.92)	0.99	(0.99 - 1.00)	0.99	(0.99 - 0.99)
**Positive test (n=506,773)**												
White	11323083	307983	1		1		1		1		1	
South Asian	1061336	50858	1.67	(1.66 - 1.69)	1.51	(1.50 - 1.53)	1.51	(1.49 - 1.52)	1.38	(1.36 - 1.39)	1.32	(1.31 - 1.33)
Black	355625	10218	1.01	(0.99 - 1.03)	0.92	(0.90 - 0.94)	0.91	(0.90 - 0.93)	0.86	(0.85 - 0.88)	0.85	(0.84 - 0.87)
Mixed	179557	5583	1.15	(1.12 - 1.18)	0.99	(0.96 - 1.01)	0.98	(0.96 - 1.01)	0.98	(0.96 - 1.01)	0.98	(0.95 - 1.01)
Other	334962	6995	0.79	(0.77 - 0.80)	0.68	(0.67 - 0.70)	0.68	(0.67 - 0.70)	0.72	(0.70 - 0.73)	0.72	(0.70 - 0.73)
Unknown	4381803	125136	1.06	(1.05 - 1.07)	1.01	(1.00 - 1.01)	1.01	(1.00 - 1.02)	1.05	(1.05 - 1.06)	1.04	(1.03 - 1.05)
**Hospital admission (n=18,855)**												
White	11323083	12120	1		1		1		1		1	
South Asian	1061336	2100	1.71	(1.62 - 1.79)	2.73	(2.60 - 2.87)	2.35	(2.24 - 2.47)	2.05	(1.95 - 2.16)	1.89	(1.79 - 2.00)
Black	355625	362	1	(0.90 - 1.11)	1.55	(1.39 - 1.73)	1.28	(1.15 - 1.43)	1.27	(1.14 - 1.41)	1.23	(1.11 - 1.37)
Mixed	179557	132	0.75	(0.63 - 0.89)	1.47	(1.23 - 1.74)	1.33	(1.12 - 1.58)	1.36	(1.14 - 1.62)	1.33	(1.12 - 1.58)
Other	334962	222	0.69	(0.60 - 0.79)	1.28	(1.12 - 1.46)	1.18	(1.03 - 1.35)	1.37	(1.20 - 1.57)	1.32	(1.16 - 1.51)
Unknown	4381803	3919	0.87	(0.84 - 0.91)	0.96	(0.93 - 1.00)	0.97	(0.93 - 1.00)	1.07	(1.04 - 1.12)	1.07	(1.03 - 1.11)
**ICU admission (n=3,351)**												
White	11323083	1969	1		1		1		1		1	
South Asian	1061336	534	2.85	(2.57 - 3.16)	4.12	(3.71 - 4.59)	3.62	(3.25 - 4.02)	2.93	(2.62 - 3.27)	2.68	(2.39 - 3.01)
Black	355625	106	1.65	(1.35 - 2.02)	2.22	(1.82 - 2.71)	1.84	(1.50 - 2.25)	1.73	(1.41 - 2.12)	1.67	(1.37 - 2.05)
Mixed	179557	44	1.43	(1.06 - 1.93)	2.45	(1.81 - 3.31)	2.21	(1.63 - 2.98)	2.3	(1.70 - 3.10)	2.23	(1.65 - 3.02)
Other	334962	60	1.05	(0.81 - 1.37)	1.74	(1.34 - 2.26)	1.6	(1.23 - 2.07)	1.97	(1.52 - 2.56)	1.9	(1.46 - 2.46)
Unknown	4381803	638	0.84	(0.77 - 0.92)	0.95	(0.86 - 1.03)	0.95	(0.87 - 1.04)	1.02	(0.93 - 1.11)	1.01	(0.92 - 1.11)

**COVID-19 death (n=7,366)**												
White	11323083	4874	1		1		1		1		1	
South Asian	1061336	532	1.17	(1.07 - 1.28)	2.8	(2.55 - 3.08)	2.35	(2.14 - 2.59)	2.19	(1.98 - 2.41)	1.87	(1.68 - 2.07)
Black	355625	75	0.52	(0.42 - 0.66)	1.17	(0.93 - 1.47)	0.94	(0.75 - 1.19)	0.94	(0.74 - 1.18)	0.92	(0.73 - 1.16)
Mixed	179557	26	0.38	(0.26 - 0.55)	1.41	(0.96 - 2.07)	1.25	(0.85 - 1.84)	1.28	(0.87 - 1.88)	1.24	(0.85 - 1.83)
Other	334962	35	0.28	(0.20 - 0.40)	0.93	(0.66 - 1.29)	0.84	(0.60 - 1.18)	0.96	(0.69 - 1.34)	0.92	(0.66 - 1.29)
Unknown	4381803	1824	1	(0.94 - 1.05)	1.06	(1.00 - 1.12)	1.07	(1.01 - 1.13)	1.16	(1.10 - 1.23)	1.17	(1.11 - 1.24)
**Non COVID-19 death (n=41,801)**												
White	11323083	28229	1		1		1		1		1	
South Asian	1061336	973	0.41	(0.38 - 0.43)	0.86	(0.81 - 0.92)	0.78	(0.73 - 0.84)	0.92	(0.86 - 0.99)	0.88	(0.82 - 0.94)
Black	355625	349	0.44	(0.40 - 0.49)	0.9	(0.81 - 1.00)	0.79	(0.71 - 0.88)	0.86	(0.77 - 0.96)	0.85	(0.77 - 0.95)
Mixed	179557	134	0.33	(0.28 - 0.39)	1.03	(0.87 - 1.22)	0.96	(0.81 - 1.14)	1.04	(0.88 - 1.23)	1.03	(0.87 - 1.23)
Other	334962	179	0.24	(0.21 - 0.28)	0.69	(0.59 - 0.79)	0.65	(0.56 - 0.76)	0.73	(0.63 - 0.85)	0.72	(0.62 - 0.84)
Unknown	4381803	11937	1.09	(1.07 - 1.11)	1.1	(1.08 - 1.13)	1.11	(1.08 - 1.13)	1.19	(1.16 - 1.21)	1.19	(1.17 - 1.22)

*Any counts <5 are set to 5 per data disclosure agreements

Table S6. Association between ethnicity in 16 categories and COVID-19 outcomes in wave 2 (serial adjustment)

	Denominator	Event	Crude		Age-Sex Adjusted		Plus Deprivation		Plus Co-morbidities and Clinical Factors		Plus Household size	
			HR	95% CI	HR	95% CI	HR	95% CI	HR	95% CI	HR	95% CI
**Tested (n=1,736,366)**												
British	9845315	1567742	1		1		1		1		1	
Irish	81185	11763	0.9	(0.88 - 0.92)	0.92	(0.90 - 0.93)	0.92	(0.90 - 0.94)	0.94	(0.92 - 0.95)	0.94	(0.92 - 0.96)
Other White	1396583	153767	0.68	(0.68 - 0.68)	0.61	(0.61 - 0.61)	0.61	(0.61 - 0.62)	0.67	(0.66 - 0.67)	0.67	(0.66 - 0.67)
White + Caribbean	41228	6868	1.03	(1.00 - 1.05)	0.9	(0.87 - 0.92)	0.9	(0.88 - 0.92)	0.9	(0.88 - 0.92)	0.9	(0.88 - 0.92)
White + African	34991	5134	0.89	(0.87 - 0.92)	0.79	(0.77 - 0.81)	0.8	(0.78 - 0.82)	0.83	(0.81 - 0.86)	0.83	(0.81 - 0.86)
White + Asian	36257	5548	0.97	(0.94 - 0.99)	0.84	(0.81 - 0.86)	0.84	(0.82 - 0.86)	0.87	(0.85 - 0.89)	0.87	(0.85 - 0.90)
Other mixed	67081	9129	0.85	(0.83 - 0.87)	0.74	(0.72 - 0.75)	0.74	(0.73 - 0.76)	0.78	(0.76 - 0.79)	0.78	(0.76 - 0.80)
Indian	447812	66056	0.92	(0.91 - 0.93)	0.86	(0.86 - 0.87)	0.87	(0.86 - 0.87)	0.88	(0.87 - 0.89)	0.88	(0.87 - 0.88)
Pakistani	309214	58882	1.16	(1.15 - 1.17)	1.03	(1.02 - 1.04)	1.04	(1.03 - 1.05)	1.02	(1.01 - 1.03)	1.02	(1.02 - 1.03)
Bangladeshi	73154	11396	0.93	(0.91 - 0.95)	0.82	(0.80 - 0.83)	0.82	(0.81 - 0.84)	0.81	(0.79 - 0.82)	0.81	(0.79 - 0.82)
Other Asian	231156	30522	0.82	(0.81 - 0.83)	0.74	(0.73 - 0.75)	0.74	(0.74 - 0.75)	0.77	(0.76 - 0.78)	0.77	(0.76 - 0.77)
Caribbean	84219	10749	0.76	(0.75 - 0.78)	0.76	(0.74 - 0.77)	0.76	(0.75 - 0.78)	0.76	(0.75 - 0.78)	0.76	(0.75 - 0.78)
African	199012	27417	0.83	(0.82 - 0.84)	0.74	(0.73 - 0.75)	0.75	(0.74 - 0.76)	0.78	(0.77 - 0.79)	0.78	(0.77 - 0.79)
Other Black	72394	9902	0.82	(0.80 - 0.84)	0.74	(0.73 - 0.76)	0.75	(0.73 - 0.76)	0.76	(0.75 - 0.78)	0.77	(0.75 - 0.78)
Chinese	105658	6444	0.37	(0.36 - 0.38)	0.32	(0.31 - 0.32)	0.32	(0.31 - 0.32)	0.37	(0.37 - 0.38)	0.38	(0.38 - 0.39)
Other	229304	26460	0.72	(0.71 - 0.72)	0.64	(0.63 - 0.65)	0.64	(0.64 - 0.65)	0.68	(0.68 - 0.69)	0.69	(0.68 - 0.70)
Unknown	4381803	639977	0.91	(0.90 - 0.91)	0.87	(0.87 - 0.87)	0.87	(0.87 - 0.87)	0.95	(0.95 - 0.95)	0.94	(0.94 - 0.95)
**Positive test (n=506,773)**												
British	9845315	275080	1		1		1		1		1	
Irish	81185	1772	0.8	(0.76 - 0.83)	0.83	(0.79 - 0.87)	0.83	(0.79 - 0.87)	0.85	(0.81 - 0.89)	0.85	(0.81 - 0.89)
Other White	1396583	31131	0.83	(0.82 - 0.84)	0.73	(0.72 - 0.74)	0.72	(0.72 - 0.73)	0.79	(0.78 - 0.79)	0.78	(0.77 - 0.79)
White + Caribbean	41228	1414	1.17	(1.11 - 1.24)	0.98	(0.93 - 1.03)	0.98	(0.93 - 1.03)	0.98	(0.93 - 1.04)	0.99	(0.94 - 1.04)
White + African	34991	1110	1.12	(1.06 - 1.19)	0.96	(0.90 - 1.01)	0.95	(0.90 - 1.01)	0.93	(0.88 - 0.99)	0.92	(0.87 - 0.98)
White + Asian	36257	1213	1.22	(1.15 - 1.29)	1.01	(0.96 - 1.07)	1.01	(0.96 - 1.07)	1.02	(0.97 - 1.08)	1.02	(0.96 - 1.08)
Other mixed	67081	1846	1.02	(0.97 - 1.07)	0.85	(0.81 - 0.89)	0.85	(0.81 - 0.88)	0.87	(0.83 - 0.91)	0.87	(0.83 - 0.91)
Indian	447812	18670	1.45	(1.43 - 1.47)	1.32	(1.30 - 1.34)	1.32	(1.30 - 1.34)	1.23	(1.21 - 1.25)	1.18	(1.16 - 1.20)
Pakistani	309214	19779	1.99	(1.96 - 2.02)	1.71	(1.69 - 1.74)	1.7	(1.68 - 1.73)	1.54	(1.52 - 1.56)	1.46	(1.44 - 1.48)
Bangladeshi	73154	3935	1.84	(1.78 - 1.89)	1.55	(1.50 - 1.60)	1.54	(1.49 - 1.59)	1.41	(1.36 - 1.45)	1.34	(1.29 - 1.38)

Other Asian	231156	8474	1.33	(1.30 - 1.36)	1.16	(1.14 - 1.19)	1.16	(1.13 - 1.18)	1.11	(1.08 - 1.13)	1.07	(1.05 - 1.10)
Caribbean	84219	2146	0.85	(0.82 - 0.89)	0.84	(0.80 - 0.88)	0.83	(0.80 - 0.87)	0.81	(0.78 - 0.85)	0.81	(0.78 - 0.85)
African	199012	5920	1.01	(0.99 - 1.04)	0.87	(0.85 - 0.90)	0.87	(0.84 - 0.89)	0.83	(0.81 - 0.85)	0.81	(0.79 - 0.83)
Other Black	72394	2152	1.01	(0.97 - 1.06)	0.88	(0.85 - 0.92)	0.88	(0.84 - 0.92)	0.85	(0.81 - 0.89)	0.84	(0.80 - 0.88)
Chinese	105658	781	0.27	(0.25 - 0.29)	0.22	(0.21 - 0.24)	0.22	(0.21 - 0.24)	0.25	(0.23 - 0.27)	0.26	(0.24 - 0.27)
Other	229304	6214	0.98	(0.96 - 1.01)	0.85	(0.83 - 0.87)	0.84	(0.82 - 0.86)	0.87	(0.85 - 0.90)	0.86	(0.84 - 0.88)
Unknown	4381803	125136	1.04	(1.03 - 1.04)	0.97	(0.96 - 0.98)	0.97	(0.96 - 0.98)	1.02	(1.02 - 1.03)	1.01	(1.00 - 1.02)
**Hospital admission (n=18,855)**												
British	9845315	11209	1		1		1		1		1	
Irish	81185	92	1.19	(0.97 - 1.47)	1.02	(0.83 - 1.25)	0.96	(0.78 - 1.17)	0.94	(0.77 - 1.16)	0.94	(0.77 - 1.16)
Other White	1396583	819	0.61	(0.57 - 0.65)	0.97	(0.91 - 1.05)	0.91	(0.84 - 0.97)	1.03	(0.95 - 1.10)	1.01	(0.94 - 1.09)
White + Caribbean	41228	39	0.81	(0.59 - 1.12)	1.5	(1.09 - 2.05)	1.3	(0.95 - 1.78)	1.27	(0.93 - 1.75)	1.27	(0.92 - 1.74)
White + African	34991	21	0.59	(0.39 - 0.91)	1.23	(0.80 - 1.89)	1.07	(0.70 - 1.64)	1.14	(0.74 - 1.75)	1.1	(0.72 - 1.69)
White + Asian	36257	34	0.9	(0.64 - 1.26)	1.93	(1.38 - 2.71)	1.84	(1.31 - 2.57)	1.94	(1.38 - 2.71)	1.88	(1.34 - 2.64)
Other mixed	67081	38	0.6	(0.43 - 0.82)	1.28	(0.93 - 1.76)	1.17	(0.85 - 1.61)	1.26	(0.91 - 1.73)	1.23	(0.89 - 1.69)
Indian	447812	665	1.31	(1.21 - 1.42)	1.94	(1.79 - 2.11)	1.76	(1.62 - 1.91)	1.66	(1.52 - 1.80)	1.57	(1.44 - 1.71)
Pakistani	309214	992	2.05	(1.92 - 2.19)	3.65	(3.41 - 3.91)	2.91	(2.72 - 3.12)	2.36	(2.20 - 2.53)	2.13	(1.98 - 2.30)
Bangladeshi	73154	151	1.83	(1.56 - 2.15)	3.72	(3.16 - 4.37)	2.96	(2.52 - 3.49)	2.55	(2.17 - 3.00)	2.31	(1.96 - 2.72)
Other Asian	231156	292	1.29	(1.14 - 1.45)	2.37	(2.11 - 2.67)	2.12	(1.88 - 2.38)	2.06	(1.83 - 2.32)	1.94	(1.72 - 2.19)
Caribbean	84219	117	1.2	(1.00 - 1.44)	1.22	(1.02 - 1.47)	1.01	(0.84 - 1.22)	0.98	(0.82 - 1.18)	0.98	(0.81 - 1.17)
African	199012	177	0.85	(0.73 - 0.98)	1.79	(1.54 - 2.08)	1.46	(1.26 - 1.70)	1.51	(1.30 - 1.76)	1.44	(1.24 - 1.68)
Other Black	72394	68	0.89	(0.70 - 1.13)	1.63	(1.28 - 2.07)	1.37	(1.08 - 1.74)	1.38	(1.09 - 1.75)	1.34	(1.05 - 1.70)
Chinese	105658	20	0.19	(0.12 - 0.29)	0.39	(0.25 - 0.61)	0.39	(0.25 - 0.60)	0.54	(0.35 - 0.84)	0.53	(0.34 - 0.82)
Other	229304	202	0.86	(0.75 - 0.99)	1.62	(1.41 - 1.87)	1.44	(1.25 - 1.66)	1.61	(1.40 - 1.86)	1.56	(1.35 - 1.79)
Unknown	4381803	3919	0.84	(0.81 - 0.87)	0.96	(0.93 - 1.00)	0.96	(0.93 - 1.00)	1.08	(1.04 - 1.12)	1.07	(1.03 - 1.11)
**ICU admission (n=3,351)**												
British	9845315	1767	1		1		1		1		1	
Irish	81185	11	0.78	(0.43 - 1.41)	0.76	(0.42 - 1.37)	0.72	(0.40 - 1.30)	0.76	(0.42 - 1.37)	0.75	(0.42 - 1.36)
Other White	1396583	191	0.81	(0.69 - 0.94)	1.27	(1.09 - 1.49)	1.18	(1.01 - 1.38)	1.39	(1.19 - 1.62)	1.36	(1.17 - 1.59)
White + Caribbean	41228	10	1.31	(0.70 - 2.45)	2.28	(1.22 - 4.25)	1.99	(1.07 - 3.71)	1.96	(1.05 - 3.65)	1.94	(1.04 - 3.62)
White + African	34991	11	1.76	(0.97 - 3.19)	2.95	(1.63 - 5.35)	2.53	(1.40 - 4.58)	2.55	(1.40 - 4.62)	2.44	(1.35 - 4.43)
White + Asian	36257	13	2.05	(1.19 - 3.53)	3.87	(2.24 - 6.69)	3.69	(2.13 - 6.37)	4.24	(2.45 - 7.33)	4.11	(2.38 - 7.10)

Other mixed	67081	10	0.86	(0.46 - 1.60)	1.65	(0.88 - 3.08)	1.5	(0.81 - 2.81)	1.68	(0.90 - 3.13)	1.63	(0.87 - 3.04)
Indian	447812	169	2.14	(1.81 - 2.52)	2.91	(2.46 - 3.45)	2.69	(2.27 - 3.19)	2.32	(1.95 - 2.76)	2.16	(1.82 - 2.58)
Pakistani	309214	211	3.47	(2.99 - 4.03)	5.76	(4.95 - 6.70)	4.63	(3.97 - 5.41)	3.44	(2.94 - 4.02)	3.1	(2.64 - 3.65)
Bangladeshi	73154	47	3.43	(2.56 - 4.60)	6.4	(4.77 - 8.59)	5.1	(3.80 - 6.86)	4.41	(3.28 - 5.94)	4	(2.96 - 5.40)
Other Asian	231156	107	2.63	(2.15 - 3.21)	4.22	(3.45 - 5.17)	3.79	(3.09 - 4.64)	3.4	(2.76 - 4.17)	3.19	(2.59 - 3.92)
Caribbean	84219	33	2.02	(1.43 - 2.87)	2.14	(1.51 - 3.04)	1.79	(1.26 - 2.54)	1.7	(1.19 - 2.41)	1.68	(1.18 - 2.39)
African	199012	51	1.39	(1.05 - 1.84)	2.3	(1.73 - 3.05)	1.87	(1.41 - 2.48)	1.81	(1.36 - 2.41)	1.72	(1.29 - 2.29)
Other Black	72394	22	1.61	(1.06 - 2.46)	2.38	(1.56 - 3.63)	2	(1.31 - 3.06)	1.96	(1.28 - 2.99)	1.9	(1.24 - 2.90)
Chinese	105658	8	0.43	(0.22 - 0.87)	0.86	(0.43 - 1.72)	0.85	(0.42 - 1.70)	1.5	(0.75 - 3.01)	1.45	(0.72 - 2.92)
Other	229304	52	1.27	(0.96 - 1.68)	2.12	(1.60 - 2.81)	1.89	(1.42 - 2.50)	2.17	(1.64 - 2.87)	2.08	(1.57 - 2.76)
Unknown	4381803	638	0.82	(0.75 - 0.90)	0.96	(0.88 - 1.06)	0.96	(0.88 - 1.05)	1.04	(0.95 - 1.15)	1.04	(0.94 - 1.14)
COVID-19 death (n=7,366)												
British	9845315	4599	1		1		1		1		1	
Irish	81185	32	1.01	(0.71 - 1.43)	0.77	(0.54 - 1.09)	0.71	(0.50 - 1.01)	0.69	(0.49 - 0.98)	0.69	(0.49 - 0.98)
Other White	1396583	243	0.43	(0.38 - 0.49)	0.97	(0.85 - 1.10)	0.92	(0.81 - 1.05)	0.97	(0.85 - 1.11)	0.96	(0.84 - 1.09)
White + Caribbean	41228	7	0.36	(0.17 - 0.76)	1.03	(0.49 - 2.17)	0.87	(0.41 - 1.82)	0.8	(0.38 - 1.69)	0.8	(0.38 - 1.69)
White + African	34991	6	0.41	(0.18 - 0.91)	1.99	(0.89 - 4.44)	1.73	(0.78 - 3.86)	2.04	(0.91 - 4.54)	1.93	(0.86 - 4.30)
White + Asian	36257	5	0.33	(0.14 - 0.80)	1.53	(0.63 - 3.67)	1.46	(0.61 - 3.52)	1.59	(0.66 - 3.83)	1.51	(0.63 - 3.64)
Other mixed	67081	8	0.31	(0.16 - 0.62)	1.41	(0.70 - 2.82)	1.28	(0.64 - 2.57)	1.38	(0.69 - 2.77)	1.35	(0.67 - 2.70)
Indian	447812	205	1.08	(0.93 - 1.24)	2.19	(1.89 - 2.54)	1.96	(1.69 - 2.27)	2.01	(1.73 - 2.33)	1.84	(1.58 - 2.15)
Pakistani	309214	240	1.35	(1.18 - 1.54)	3.77	(3.30 - 4.31)	2.88	(2.51 - 3.29)	2.42	(2.11 - 2.78)	1.94	(1.67 - 2.24)
Bangladeshi	73154	39	1.16	(0.85 - 1.60)	4.57	(3.33 - 6.28)	3.48	(2.53 - 4.78)	2.84	(2.07 - 3.91)	2.26	(1.64 - 3.13)
Other Asian	231156	48	0.54	(0.40 - 0.72)	1.76	(1.32 - 2.34)	1.55	(1.16 - 2.07)	1.58	(1.18 - 2.11)	1.41	(1.05 - 1.88)
Caribbean	84219	44	1.12	(0.83 - 1.51)	1.1	(0.82 - 1.49)	0.89	(0.66 - 1.20)	0.85	(0.63 - 1.14)	0.85	(0.63 - 1.15)
African	199012	19	0.22	(0.14 - 0.35)	1.16	(0.74 - 1.83)	0.93	(0.59 - 1.47)	1.03	(0.65 - 1.62)	0.95	(0.61 - 1.50)
Other Black	72394	12	0.38	(0.22 - 0.67)	1.31	(0.74 - 2.31)	1.08	(0.61 - 1.91)	1.12	(0.64 - 1.98)	1.08	(0.61 - 1.90)
Chinese	105658	6	0.14	(0.06 - 0.32)	0.54	(0.24 - 1.20)	0.52	(0.23 - 1.16)	0.72	(0.32 - 1.59)	0.7	(0.31 - 1.55)
Other	229304	29	0.31	(0.22 - 0.45)	1.06	(0.73 - 1.53)	0.94	(0.65 - 1.36)	1.02	(0.70 - 1.47)	0.97	(0.67 - 1.40)
Unknown	4381803	1824	0.94	(0.89 - 0.99)	1.05	(1.00 - 1.11)	1.06	(1.00 - 1.12)	1.16	(1.10 - 1.23)	1.16	(1.10 - 1.23)
Non COVID-19 death (n=41,801)												
British	9845315	26499	1		1		1		1		1	
Irish	81185	289	1.45	(1.29 - 1.63)	1.18	(1.05 - 1.33)	1.13	(1.00 - 1.26)	1.05	(0.94 - 1.18)	1.05	(0.93 - 1.18)

Other White	1396583	1441	0.41	(0.39 - 0.44)	0.9	(0.86 - 0.95)	0.87	(0.83 - 0.92)	0.91	(0.86 - 0.96)	0.91	(0.86 - 0.96)
White + Caribbean	41228	47	0.45	(0.34 - 0.60)	1.15	(0.86 - 1.53)	1.04	(0.78 - 1.38)	1.07	(0.80 - 1.42)	1.07	(0.80 - 1.42)
White + African	34991	21	0.24	(0.16 - 0.37)	0.93	(0.61 - 1.43)	0.85	(0.56 - 1.31)	1.05	(0.68 - 1.60)	1.03	(0.67 - 1.57)
White + Asian	36257	23	0.25	(0.17 - 0.38)	0.95	(0.63 - 1.43)	0.93	(0.61 - 1.39)	0.98	(0.65 - 1.47)	0.97	(0.64 - 1.46)
Other mixed	67081	43	0.26	(0.20 - 0.36)	0.98	(0.72 - 1.32)	0.93	(0.69 - 1.25)	1.02	(0.75 - 1.37)	1.01	(0.75 - 1.36)
Indian	447812	488	0.44	(0.41 - 0.49)	0.84	(0.77 - 0.92)	0.79	(0.72 - 0.86)	0.97	(0.88 - 1.06)	0.94	(0.86 - 1.03)
Pakistani	309214	314	0.4	(0.35 - 0.44)	1.03	(0.92 - 1.15)	0.87	(0.78 - 0.98)	0.96	(0.86 - 1.08)	0.88	(0.79 - 0.99)
Bangladeshi	73154	53	0.29	(0.22 - 0.38)	1	(0.76 - 1.31)	0.85	(0.65 - 1.11)	0.85	(0.65 - 1.12)	0.79	(0.60 - 1.04)
Other Asian	231156	118	0.22	(0.18 - 0.26)	0.59	(0.49 - 0.71)	0.55	(0.46 - 0.67)	0.68	(0.57 - 0.82)	0.66	(0.55 - 0.79)
Caribbean	84219	185	0.9	(0.78 - 1.04)	0.89	(0.77 - 1.03)	0.78	(0.67 - 0.90)	0.8	(0.69 - 0.92)	0.8	(0.69 - 0.93)
African	199012	103	0.21	(0.18 - 0.26)	0.83	(0.68 - 1.01)	0.73	(0.60 - 0.89)	0.9	(0.74 - 1.09)	0.88	(0.72 - 1.07)
Other Black	72394	61	0.34	(0.27 - 0.44)	1.02	(0.79 - 1.31)	0.91	(0.71 - 1.17)	0.96	(0.75 - 1.24)	0.95	(0.74 - 1.22)
Chinese	105658	46	0.18	(0.13 - 0.23)	0.62	(0.46 - 0.83)	0.61	(0.45 - 0.81)	0.74	(0.56 - 0.99)	0.74	(0.55 - 0.99)
Other	229304	133	0.24	(0.20 - 0.28)	0.7	(0.59 - 0.83)	0.66	(0.55 - 0.78)	0.72	(0.60 - 0.85)	0.71	(0.60 - 0.84)
Unknown	4381803	11937	1.02	(1.00 - 1.04)	1.1	(1.08 - 1.12)	1.1	(1.08 - 1.12)	1.18	(1.16 - 1.21)	1.19	(1.16 - 1.21)

*Any counts <5 are set to 5 per data disclosure agreements

Table S7. Testing the proportional Hazards assumptions for each outcome (wave 1)

Testing the PH assumption for each outcome by ethnic group	Global p.value		
	Crude	Age/Sex Adjusted	Fully Adjusted
	p-value	p-value	p-value
tested	0.905	0.699	0.59
positivetest	0.225	0.25	0.293
icu	0.959	0.828	0.854
hes	0.167	0.063	0.102
onscoviddeath	0.885	1	1
ons_noncoviddeath	0.527	0.173	0.222
onsdeath	0.529	0.201	0.257

Figure S1. Odds of testing positive for SARS-CoV-2 amongst those ever receiving a test

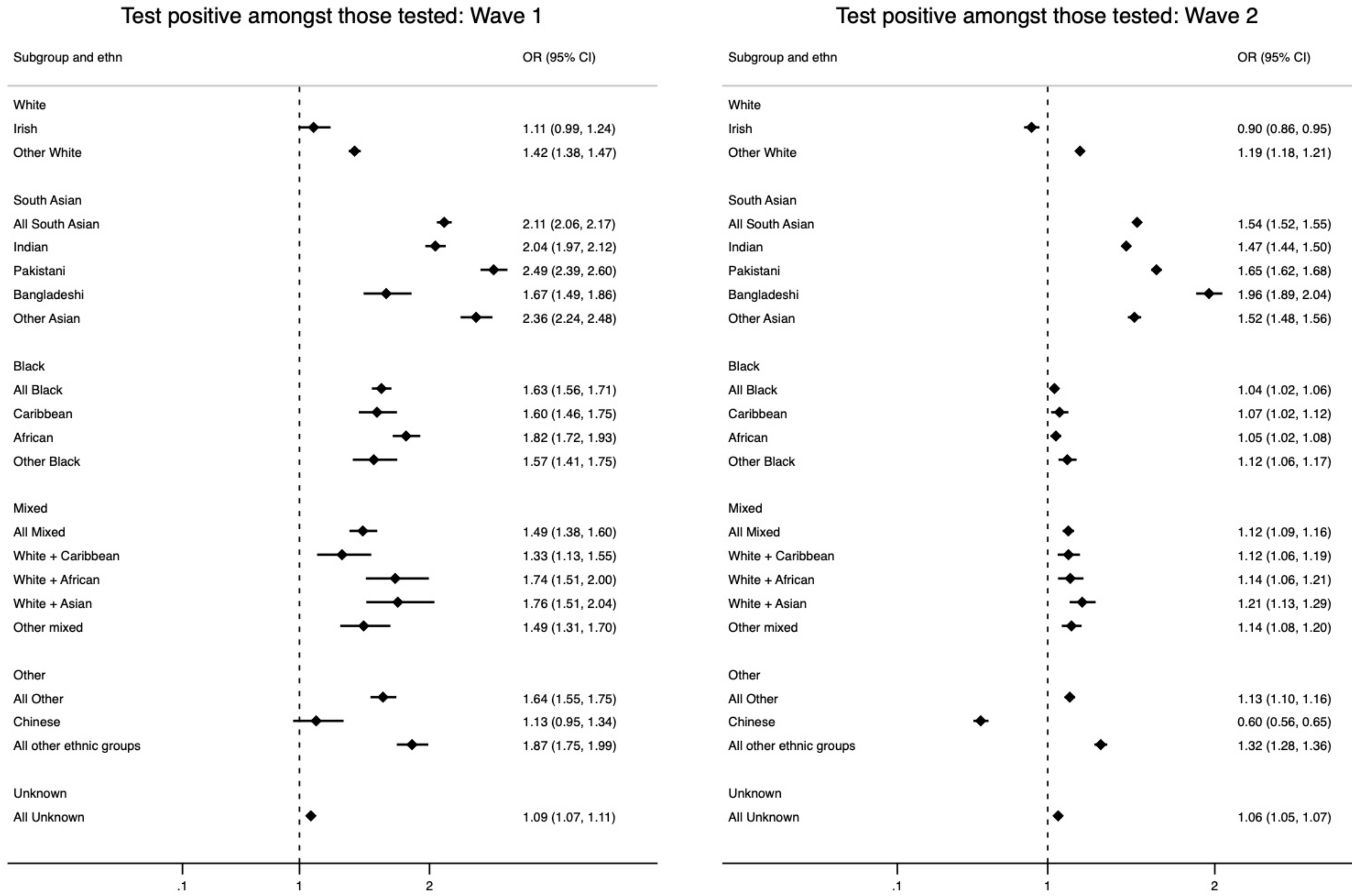

*All White is the reference category for comparison of ethnicity in 5 categories. British is the reference category for comparison of ethnicity in 16 categories. Models adjust for age, sex, deprivation quintile, all pre-specified clinical co-morbidities, categories of BMI, HbA1c, and systolic and diastolic blood pressure, number of primary care consultations in the 12 months prior, and STP region.

Figure S2. Association between ethnicity in 5 categories and COVID-19 outcomes in the care-home population in wave 1 and wave 2

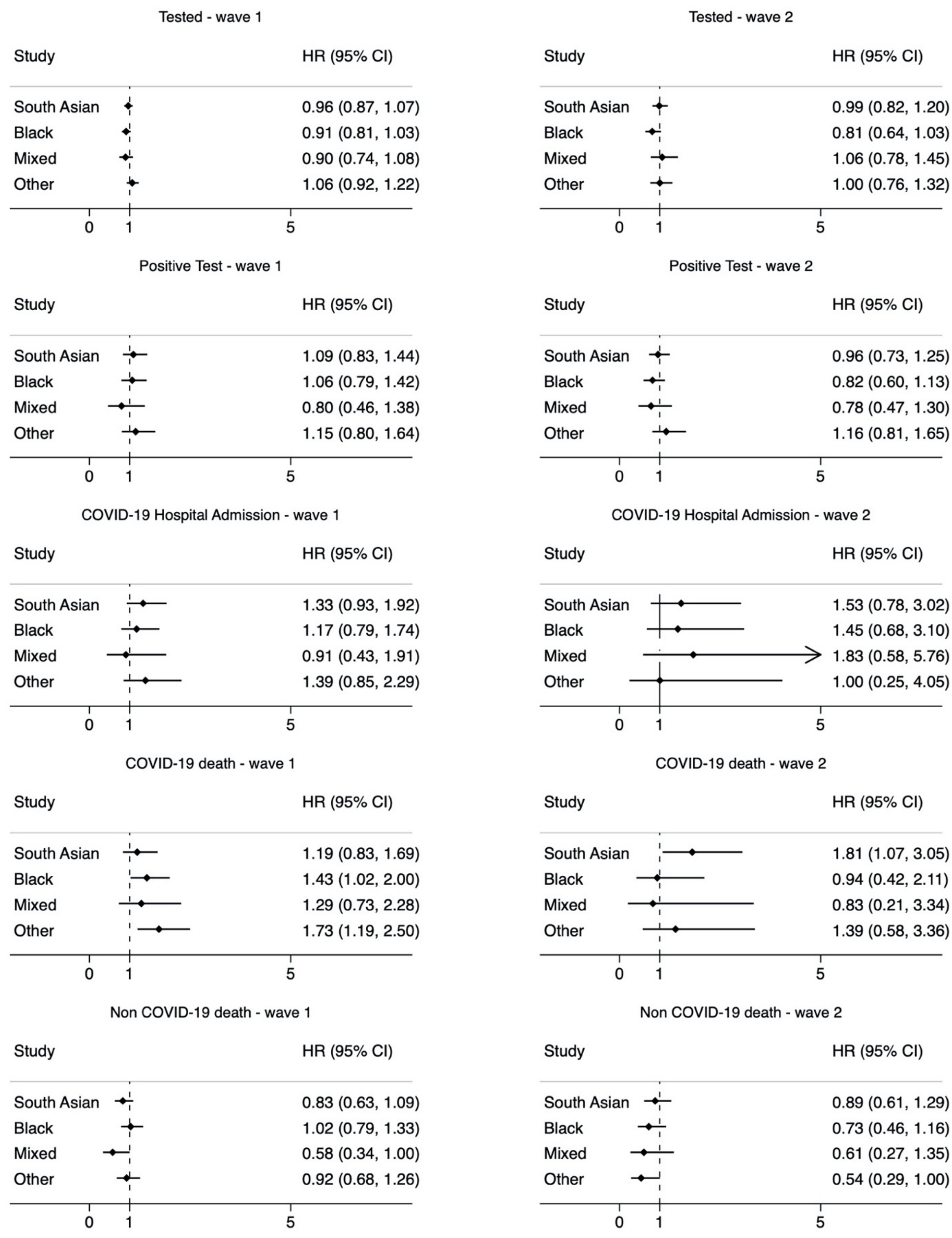

*White is the reference category. Models adjust for age, sex, deprivation quintile, all pre-specified clinical co-morbidities, categories of BMI, HbA1c, and systolic and diastolic blood pressure, number of primary care consultations in the 12 months prior, and stratification by STP region.

Figure S3. Ethnic differences in all study outcomes in wave 1: Complete case analysis (CC) vs. multiple imputation (MI) in wave 1

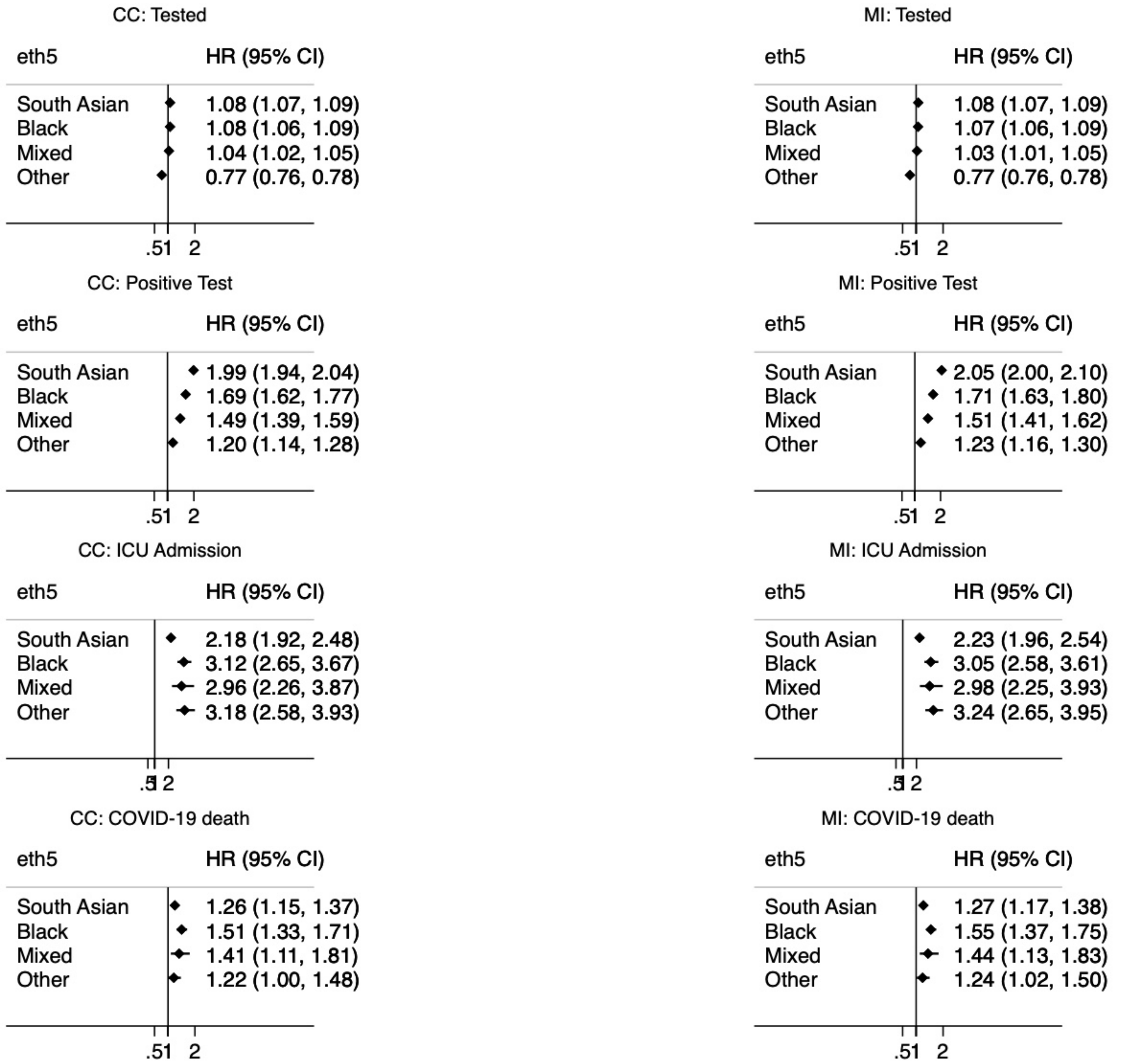

*All White is the reference category. Models adjust for age, sex, deprivation quintile, all pre-specified clinical co-morbidities, categories of BMI, HbA1c, and systolic and diastolic blood pressure, number of primary care consultations in the 12 months prior, household size, and stratification by STP region.

*Multiple imputation of missing ethnicity was performed in Stata using the mi suite of commands. Ethnicity data were considered to be missing at random. Multinomial logistic regression was used as the imputation model, including all other explanatory variables and the binary outcome indicator from the substantive regression in the model. 10 imputed datasets were created. Hazard ratios were combined using Rubin's rules.

Figure S4. Comparison between COVID-19 as any cause of death vs. underlying cause of death in wave 1

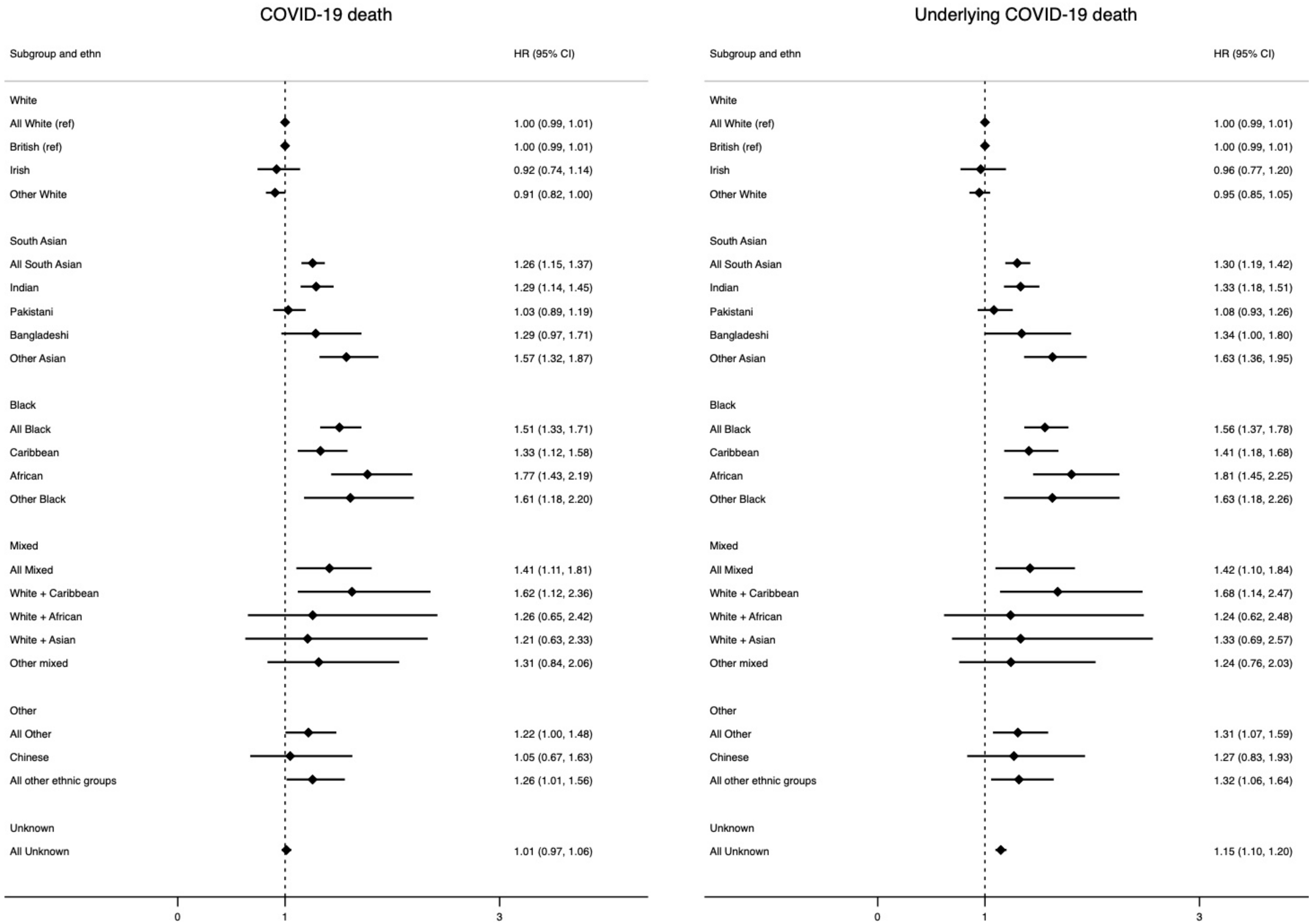

*All White is the reference category for comparison of ethnicity in 5 categories. British is the reference category for comparison of ethnicity in 16 categories. Models adjust for age, sex, deprivation quintile, all pre-specified clinical co-morbidities, categories of BMI, HbA1c, and systolic and diastolic blood pressure, number of primary care consultations in the 12 months prior, and stratification by STP region.

Figure S5. Ethnic differences in COVID-19 outcomes with and without clustering by STP region in wave 1

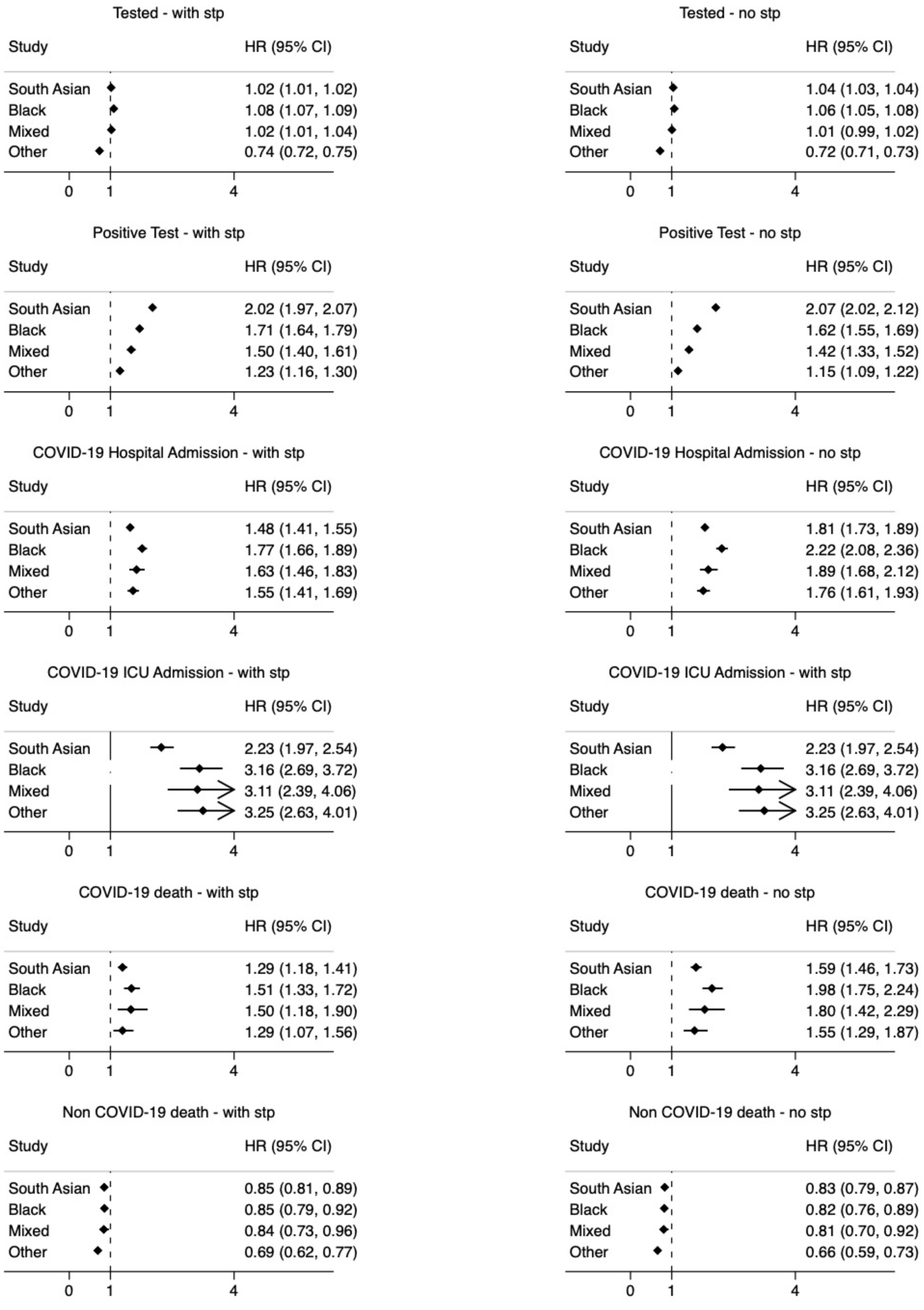

*White is the reference category. *models adjust for age, sex, deprivation quintile, all pre-specified clinical co-morbidities, categories of BMI, HbA1c, and systolic and diastolic blood pressure, number of primary care consultations in the 12 months prior.

Figure S6. Kaplan Meier Plots for survival by ethnic group and outcome in wave 1

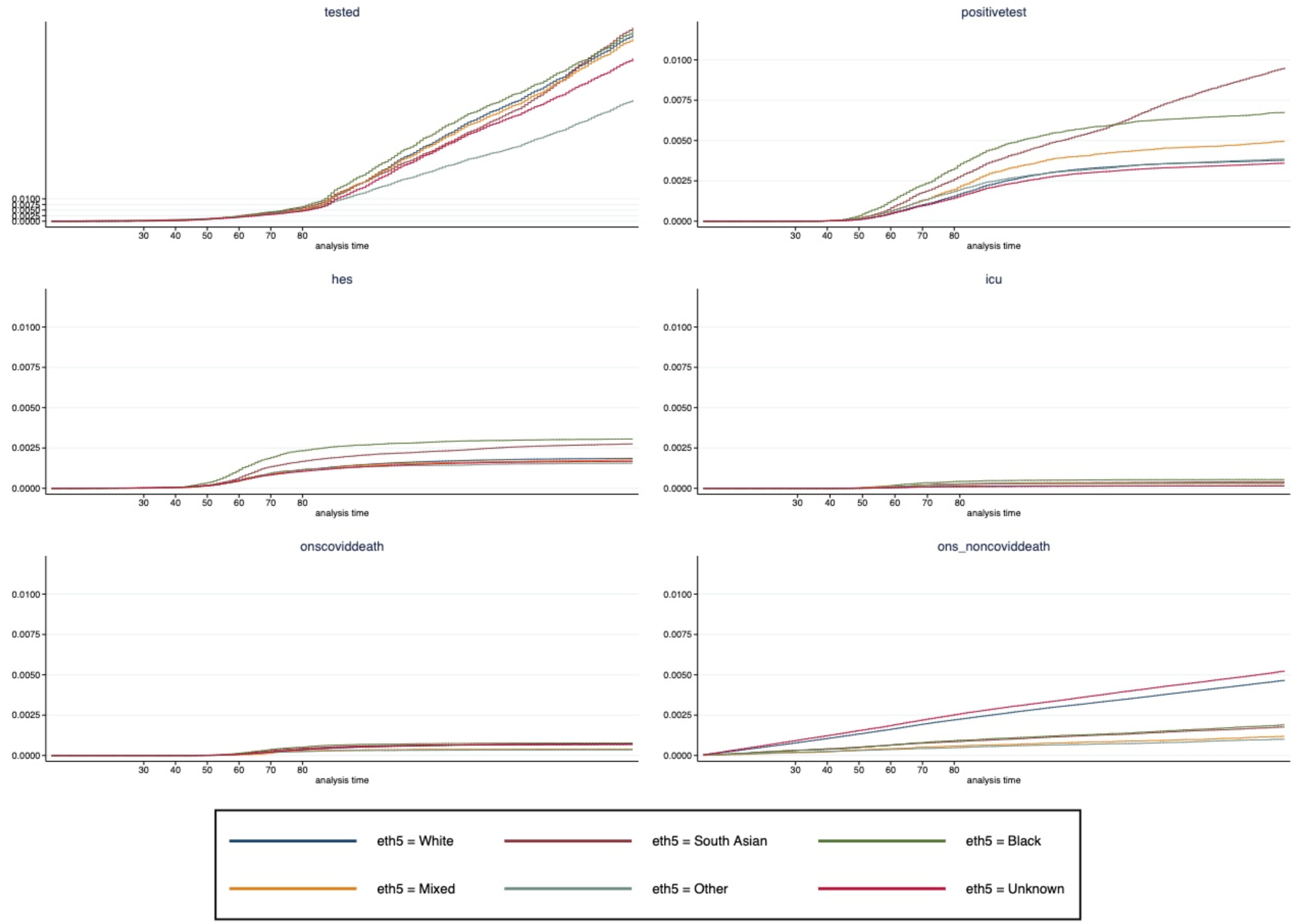

Data access and verification

Access to the underlying identifiable and potentially re-identifiable pseudonymised electronic health record data is tightly governed by various legislative and regulatory frameworks, and restricted by best practice. The data in OpenSAFELY is drawn from General Practice data across England where TPP is the Data Processor. TPP developers (CB, RC, JP, FH, and SH) initiate an automated process to create pseudonymised records in the core OpenSAFELY database, which are copies of key structured data tables in the identifiable records. These are linked onto key external data resources that have also been pseudonymised via SHA-512 one-way hashing of NHS numbers using a shared salt. DataLab developers and PIs (BG, LS, CEM, SB, AJW, WH, DE, PI, and CTR) holding contracts with NHS England have access to the OpenSAFELY pseudonymised data tables as needed to develop the OpenSAFELY tools. These tools in turn enable researchers with OpenSAFELY Data Access Agreements to write and execute code for data management and data analysis without direct access to the underlying raw pseudonymised patient data, and to review the outputs of this code. All code for the full data management pipeline—from raw data to completed results for this analysis—and for the OpenSAFELY platform as a whole is available for review at github.com/OpenSAFELY. The data management and analysis code for this paper was led by RM, CTR, and CEM and contributed to by WJH, AS, BM, RME, KB, AYSW, EJW, HF, KW, HIM, CB, SB, AJW, AM, HJC, NJD, IDJ, LT, SJWE, and LS.
